# Supplementary material for: Live-dead assay on unlabeled cells using phase imaging with computational specificity
Source: Nat Commun. 2022 Feb 7;13:713. doi: 10.1038/s41467-022-28214-x (PMC8821584; doi:10.1038/s41467-022-28214-x)
Supplement: Supplementary file 1 — Supplementary Information [file 41467_2022_28214_MOESM1_ESM.pdf]

Supplemental Information for

Live-dead assay on unlabeled cells using phase imaging with computational specificity

Chenfei Hu, Shenghua He, Young Jae Lee, Yuchen He, Edward M. Kong, Hua Li

Mark A. Anastasio, and Gabriel Popescu

Supplementary Note 1: Semantic map generation

Semantic segmentation maps were generated in MATLAB with a customized script. First, for each NucBlue and NucGreen image pair, an adaptive thresholding was applied to separate the cell nucleus and background, where the segmented cell nuclei were obtained by computing the union of the binarized fluorescent image pair. We removed the segmentation artifacts by filtering out the tiny objects below the size of a typical nucleus. Next, using on the segmentation masks, we calculated the ratio between the NucGreen and NucBlue fluorescence signal. A histogram of the average ratio within the cell nucleus is plotted in Fig. S1, where three distinctive peaks were observed corresponding to the live, injured and dead cells. Because NucGreen/NucBlue reagent is only designed for live and dead classification, the histogram of injured cells is partially overlapped with the live cells. By selecting a threshold value that gives the lowest histogram count between dead and injured cells, we assigned label "live" to all live and injured cells, while the remaining cells as "dead".

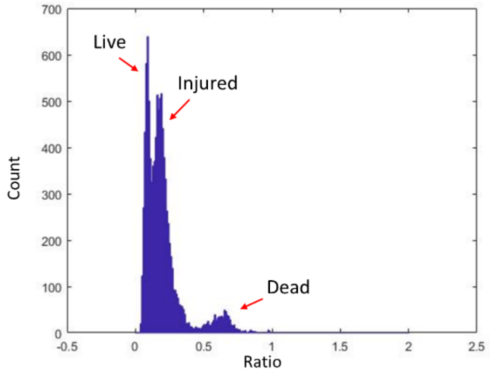

Figure S1. Histogram of fluorescence signal ratio.

Supplementary Note 2: EfficientNet

The MBConvX is the principal module in an EfficientNet. It approximately factorizes a standard convolutional layer into a sequence of separable layers to shrink the number of parameters needed in a convolution operation while maintaining a comparable ability of feature extraction. The separable layers in a MBConvX module are shown in Fig. 2c. Here, MBConv1(X=1) and MBConv6 (X=6) indicate that a ReLU layer and ReLU6 layer are employed in this module, respectively. ReLU6 is a modification of the rectified linear unit, where the activation is limited to a maximum size of 6. A MBConvX module in Fig. 2b may include a down-sampling layer, which can be inferred by the indicated feature map dimensions. The first MBConvX in each layer block does not contain a skip connection between its input and output (indicated as a dash line in Fig. 2c), since the input and output of that module have different sizes.

Supplementary Note 3: PICS evaluation at a cellular level

We implemented a U-Net based EfficientNet (E-U-Net) to extract markers associated with viable state of cells measured by SLIM. In Table S1, we show the conventional confusion matrix and corresponding F1 score evaluated on pixels in testing images. Figure S2a shows a represented raw E-U-Net output image. As indicated by the yellow arrow, there exist cases where a segmented cell may have multiple semantic labels. The conventional deep learning evaluation method only focuses on assessing pixel-wise segmentation accuracy, which overlooks some biologically relevant instances (the viable state of the entire cell) [1]. And this motives us to adopt an object-based evaluation that estimates the E-U-Net accuracy for individual cell.

Actual<br>Prediction	Live	Dead	Background
**Live**	3699117	2591	1068559
**Dead**	1422	221396	187715
**Background**	696478	44028	62668870
**F1 Score**	80.7%	65.3%	98.4%

Table S1. **Pixel-wise evaluation of the trained E-U-Net.** Due to the fact that the E-U-Net prediction assigns multiple labels to one cell nucleus, we converted the pixel-wise classification into cell-wise classification, which is more relevant biologically (Table 1 in the main text).

First, we use dominant semantic label across a cellular region to denote the viable state for this cell (Figure S2b). And we compare this semantic label with the same cell in ground truth image, repeat this step across all testing images, and obtain the cell-wise evaluation as shown in the article Table 1.

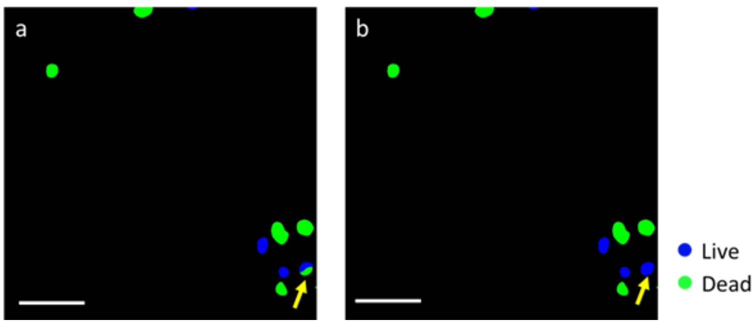

Figure S2. **a.** Output of E-U-Net on a representative testing image. The network assigns semantic labels to each pixel, and thus for some cells, more than one semantic label can be observed within the cell body. **b.** we use the dominant semantic label to indicate the viability state of a cell, and then the performance of training is evaluated at a cellular level, referred to the cell-wise evaluation. The images are randomly selected from a combined dataset across 4 imaging experiments. Scale bars: 50 µm in space.

Supplementary Note 4: PICS on CHO cells

Evaluate the effect of lytic cell death

Before performing experiments on CHO cells, a preliminary study was conducted, as follows. We prepared live cell cultures and split them into the two groups. 1 µM of staurosporine was added into the medium of the experimental group, whereas the others were kept intact as control. Both control and experimental cells were measured with SLIM for 10 hours under regular incubation condition (37 °C and 5% concentration of CO_2). Figure S3a and S3b show the QPI images of experimental and control cells measured at t = 0.5, 6.5, 7 and 10 hours, respectively. Throughout the time-course, the untreated cells remained attached to the petri-dish. Moreover, as indicated by the yellow arrows, the control cells divided at t = 6.5 hr. In contrast, cells treated with staurosporine

presented drastically different characteristics, where the cell volume decreased, and membrane ruptured or became detached. This preliminary result suggests that, under our regular incubation condition, the cells did not suffer from lytic cell death.

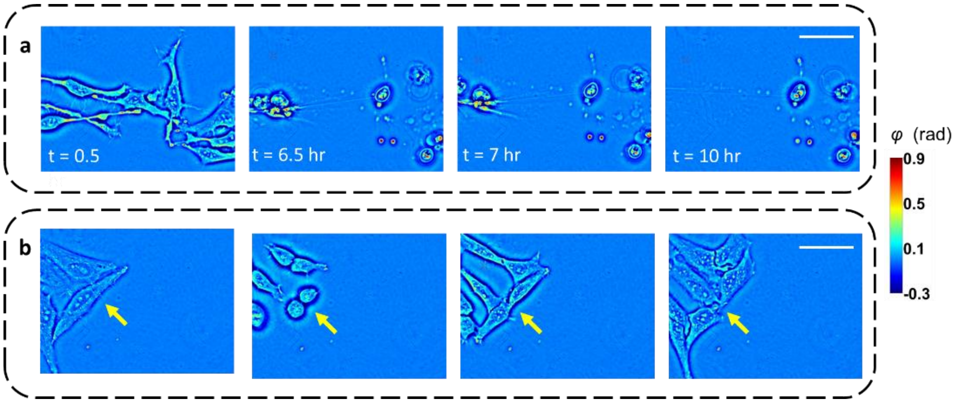

Figure S3. Time-lapse SLIM recording of CHO cells with (**a**) and without (**b**) staurosporine that introduces cell apoptosis, under regular incubation condition. For the control group, the cells continued growing and dividing without signs of cell death, which ruled out the existence of lytic cell death. The images are selected from 1 experiments, and the results are consistent across 27 measured field of views (FOV). Scale bar: 50 µm in space

PICS training and testing on CHO cell images

After validation the efficacy of staurosporine on introducing apoptotic cell death, we acquired images on CHO cells and generated the dataset for PICS training. The training was conducted on E-U-Net (EfficientNet-B7), whose network architecture, and its training/validation loss are shown in Figure. S4.

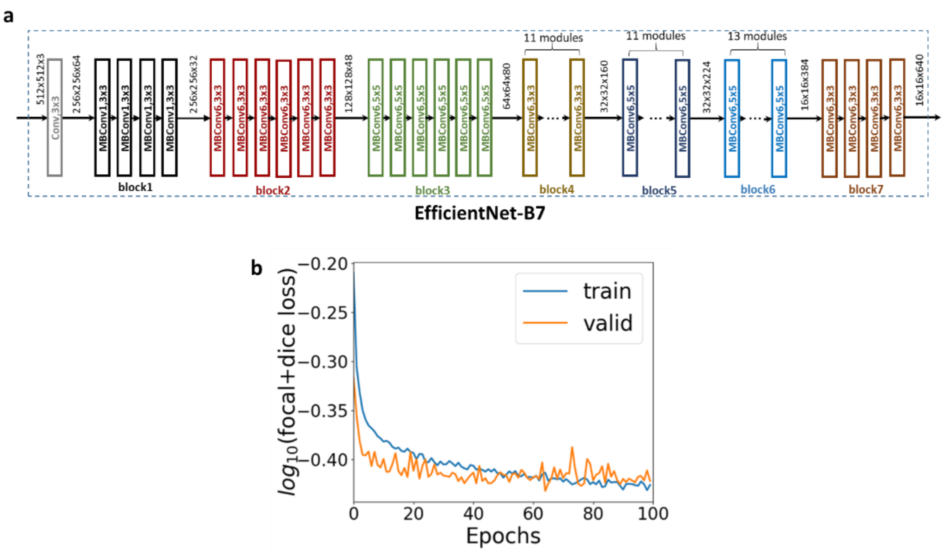

Figure S4. **CHO cells viability training with EfficientNet-B7. a.** The network architecture of EfficientNet-B7. **b.** Training and validation focal losses vs number of epochs plotted in the log scale.

We visually inspected the difference between the ground truth and machine learning prediction in the testing dataset. First, we saw prediction errors due to cells located at the boundary of the FOV, as explained in the previous comments. In addition, we found rare cases where live CHO cells were mistakenly labeled as dead (see Fig. S5 below for an illustration of CHO cells with staurosporine administration at t = 0.5 hour). In SLIM, these cells present features of abnormal cell shapes and decreased phase values, but severe membrane rupture was not observed. Previous studies suggested that these morphological features are early indicators of cell death [2-4], but it was identified as live using traditional fluorometric evaluation.

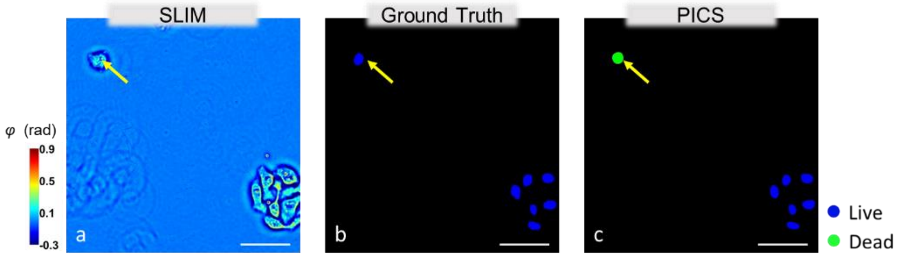

Figure S5. Cells with irregular shapes but no severe membrane rupture are subjected to erroneous classification. **a**. Input SLIM image. **b**. Ground truth. **c**. PICS output based on input in **a**. The images are randomly selected from a combined dataset across 4 imaging experiments. Scale bar: 50 μm in space

PICS performance on cells under different confluence

As discussed in the manuscript, live CHO cell culture was prepared in a 6-well plate at three confluence levels, staurosporine solution was added into the culture medium to introduce apoptosis. Figure. S6 show SLIM image of high, intermediate, and low confluence CHO cells measured at t = 0. Although, aggregating into clusters, the cell shape and boundary can be easily identified. All SLIM images were combined for training and validation. In testing, we estimated the PICS performance vs. cell confluence, and the results are summarized in Table. S2a-c.

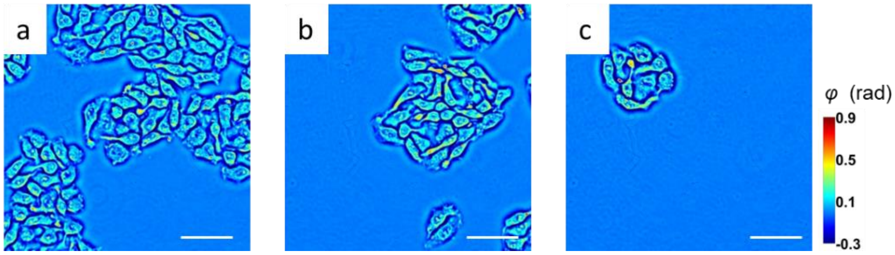

Figure S6. SLIM images of high (a), intermediate (b), and low (c) confluence CHO cells. The images are randomly selected from a combined dataset across 4 imaging experiments. Scale bar: 50 μm in space

a

High	Live	Dead
Precision	90.5%	98.5%
Recall	94.7%	97.2%
F1 Score	92.6%	97.8%

b

Intermediate	Live	Dead
Precision	87.5%	98.7%
Recall	96.3%	95.2%
F1 Score	91.7%	96.9%

c

Low	Live	Dead
Precision	96.2%	97.1%
Recall	90.1%	98.9%
F1 Score	93.1%	97.9%

Table S2. PICS performance vs. CHO cell confluence

Training on unlabeled cell SLIM images

During the data acquisition, we added FL viability reagents at the beginning, and this allows us to monitor the viable state changes of the individual cells over time. However, such data

acquisition strategy can, in principle, introduce bias when optimizing the E-U-Net. This effect can be ruled out by collecting label-free images first, followed by exogenous staining and fluorescent imaging to obtain the ground truth, at the cost of increased efforts in staining, selecting FOV and re-focusing.

To study this potential effect, we performed a control experiment described as follows. Live CHO cells were prepared and passaged onto two glass-bottom 6-well plates. 1 μM of staurosporine was added into each well to introduce apoptosis. At t = 0, cells in one well were imaged by SLIM, followed by reagents staining and fluorescence imaging. After 60 minutes, we repeated this step, but measuring the cells in the other well. Throughout the experiment, the cells were maintained in 37 °C and 5% concentration of CO_2. In this way, cells in each well were only measured once, and we obtained a dataset of unlabeled QPI images that resemble the structure of a testing dataset used in this study. The experiment was repeated 4 times, resulting in a total of 2400 SLIM and fluorescent pairs, on which PICS training and testing were performed. Table S3 shows the PICS performance on this new dataset, where live and dead cells were classified with 99% and 97% sensitivity, respectively. Thus, we can conclude that PICS optimization on cells without fluorescent stains does not compromise the prediction accuracy, which makes the proposed live-dead assay method robust for a variety of experiment settings.

Actual<br>Prediction	Live (n= 2707)	Dead (n = 101)
**Live**	98.9%	3.5%
**Dead**	1.1%	96.5%
**Precision**	99.8%	78.6%
**Recall**	98.9%	96.5%
**F1 Score**	99.3%	86.6%

Table S3. Evaluation of the PICS performance on truly unlabeled CHO cells with apoptosis reagents.

Supplementary Note 5: Comparison of PICS performance under various training strategies

We have attempted to compare cell viability prediction performance under various network architecture settings. We compared three network settings: 1) an E-U-net trained by use of a pre-trained EfficientNet; 2) an E-U-net trained from scratch; and 3) a standard U-net [5] trained from scratch. In these additional experiments, the U-net architecture employed was a standard U-net [5], with the exception that batch normalization layers were placed after each convolutional layer to facilitate the network training. EfficientNet-B0 was employed in the E-U-nets to make sure that the network size of E-U-net (7.8 million of parameters) approximately matched that of a standard U-net (7.85 million of parameters). A combined loss that comprised focal and dice losses (denoted as dice+focal loss) was used for network training. Other training settings were consistent with how the E-U-net was trained, as described in the manuscript. After the networks were trained with training and validation data from HeLa cell datasets and CHO cell datasets, they were tested on

the testing data from the two datasets, respectively. The average pixel-wise F1 scores over the live, dead and background classes were computed to evaluate the performance of the trained networks, as reported in Table S4. It can be observed from the table that, on both the two testing datasets, the average F1 scores corresponding to an E-U-net are much higher than those corresponding to a standard U-net when both of them were trained from scratch. Furthermore, as expected, an E-U-net trained with a pre-trained EfficientNet achieves a better performance than the one trained from scratch. These results demonstrate the effectiveness of the E-U-net architecture and the transfer learning techniques in training a deep neural network for pixel-wise cell viability prediction.

Network	E-U-Net (pre-trained)	E-U-Net (scratch)	Standard U-Net (scratch)
**HeLa**	80.6%	75.0%	72.0%
**CHO**	85.7%	80.4%	76.0

Table S4. Average F1 scores related to E-U-nets trained with a pre-trained EfficientNet-B0, E-U-nets trained from scratch, and standard U-nets trained from scratch, respectively.

In addition, we compared the average pixel-wise F1 scores corresponding to E-U-nets trained with various loss functions, including a dice+focal loss, a standard focal loss, a standard dice loss, and a weighted cross entropy (WCE) loss. To be consistent with the network settings in the manuscript, a pre-trained EfficientNet-B3 and a pre-trained EfficientNet-B7 were employed for training the E-U-nets on the HeLa cell dataset and CHO cell datasets, respectively. The class weights related to live, dead, and background classes in the weighted cross entropy loss were set to [0.17, 2.82, 0.012] and [2.32, 0.654, 0.027] for the network training on the HeLa cell dataset and CHO cell datasets, respectively. In each of the weight cross entropy losses, the average of weights over the three classes is 1, and the weights related to each class were inversely proportional

to the percentages of pixels from each class in the HeLa cell and CHO cell training datasets: [6.7%, 0.4%, 92.9%] and [1.1%, 3.9%, 95%], respectively. Other network training settings were consistent with how the E-U-net was trained as described in the manuscript. The trained networks were then evaluated on the testing HeLa cell dataset containing 100 images and testing CHO cell dataset containing 288 images, respectively. The average pixel-wise F1 scores were computed over all pixels in the two testing sets as shown in Table S5. It can be observed in the table that, on both the two datasets, E-U-nets trained with a dice+focal loss produced higher average pixel-wise F1 scores than those trained with a dice loss or a WCE loss.

Loss	Dice + focal	Focal	Dice	WCE
HeLa	81.2%	81.4%	80.8%	79.4
CHO	88.0%	87.4%	87.0%	85.2%

Table S5. Average F1 scores related to E-U-nets trained with various loss functions.

We further compared E-U-nets trained with a dice+focal loss to those trained with a dice loss or a WCE loss by investigating their agreements on the dice coefficients of each class related to the predictions for each image sample in the two testing datasets. Here, let us denote $D_{\text{dice+focal}}$, D_{dice}, and D_{WCE} as the dice coefficients produced by E-U-nets trained with a dice+focal loss, a dice loss and a weighted cross entropy loss, respectively. Bland-Altman plots were employed to analyze the agreement between $D_{\text{dice+focal}}$ and D_{dice} and that between $D_{\text{dice+focal}}$ and D_{WCE} on testing dataset of HeLa and that of CHO, respectively. Here, a Bland-Altman plot of two paired dice coefficients (i.e. $D_{\text{dice+focal}}$ vs. D_{dice}) produces a scatter plot x-y, in which the y axis (vertical axis) represents the difference between the two paired dice coefficients (i.e. $D_{\text{dice+focal}}$ - D_{dice}) and the x axis (horizontal

axis) shows the average of the two dice coefficients (i.e. ($D_{\text{dice+focal}}$ + D_{dice})/2). μ_d and σ_d represent the mean and standard deviation of the differences of the paired dice coefficients over the image samples in a specific testing dataset. The results corresponding to $D_{\text{dice+focal}}$ vs. D_{dice} and $D_{\text{dice+focal}}$ vs. D_{WCE} are reported in Figure S7 and Figure S8, respectively. In each figure, the subplots from left to right show the Bland-Altman plots related to the predictions for live, dead, and background classes, respectively. It can be observed from Figures S7-8 that, for predicting live and dead pixels, both the $D_{\text{dice+focal}} > D_{\text{dice}}$ (or $D_{\text{dice+focal}}$ - $D_{\text{dice}} > 0$) and $D_{\text{dice+focal}} > D_{\text{WCE}}$ (or $D_{\text{dice+focal}} - D_{\text{WCE}} > 0$) hold at the majority of the image samples in the two datasets, though for the background prediction, $D_{\text{dice+focal}}$ is comparable to D_{dice} and D_{WCE}. These results suggest that compared to a dice or WCE loss, a focal+dice loss can improve the performance of predicting live and dead pixels for the majority of testing images from both the two datasets.

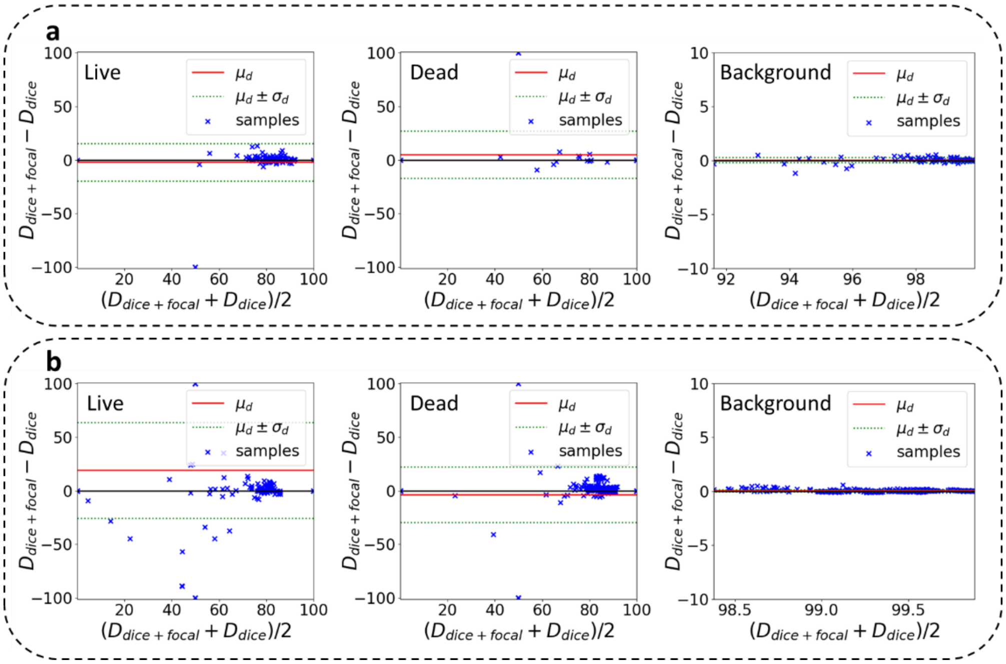

Figure S7. $D_{\text{dice+focal}}$ vs. D_{dice} on testing dataset of HeLa (**a**) and CHO (**b**), where μ_d and σ_d represent the mean and standard deviation of $D_{\text{dice+focal}}$ - D_{dice} .

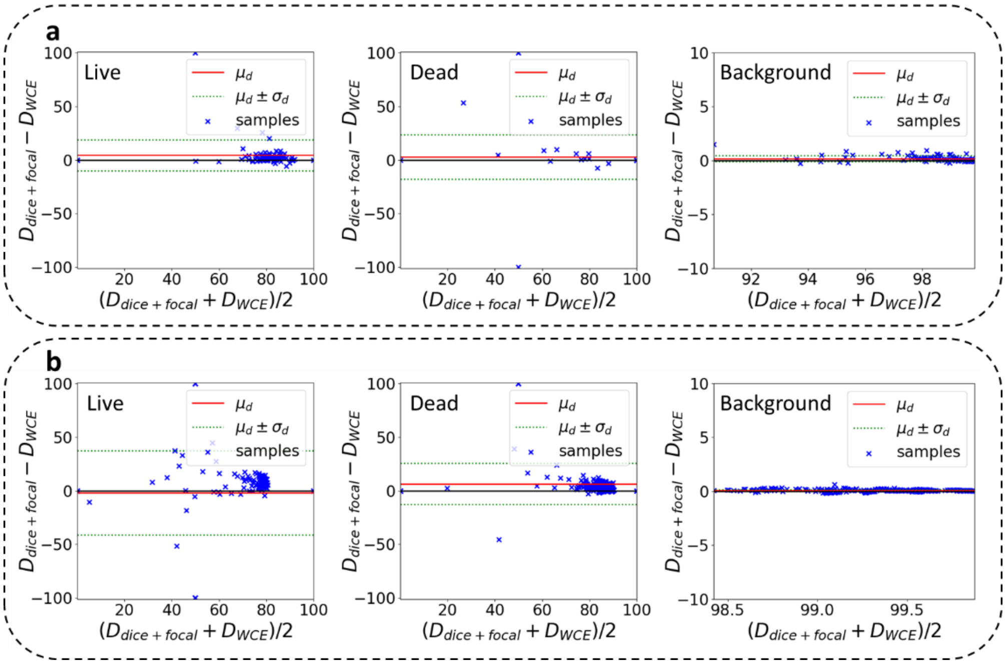

Figure S8. $D_{\text{dice+focal}}$ vs. D_{WCE} on testing dataset of HeLa (**a**) and CHO (**b**), where μ_d and σ_d represent the mean and standard deviation of $D_{\text{dice+focal}}$ - D_{WCE} .

References:

1. Caicedo, J.C., et al., *Evaluation of Deep Learning Strategies for Nucleus Segmentation in Fluorescence Images.* Cytometry A, 2019. **95**(9): p. 952-965.
2. Eldridge, W.J., J. Hoballah, and A. Wax, *Molecular and biophysical analysis of apoptosis using a combined quantitative phase imaging and fluorescence resonance energy transfer microscope.* Journal of Biophotonics, 2018. **11**(12): p. e201800126.
3. Pavillon, N., et al., *Early cell death detection with digital holographic microscopy.* PLoS One, 2012. **7**(1): p. e30912.
4. Vicar, T., et al., *The Quantitative-Phase Dynamics of Apoptosis and Lytic Cell Death.* Scientific Reports, 2020. **10**(1).
5. Ronneberger, O., P. Fischer, and T. Brox, *U-Net: Convolutional networks for biomedical image segmentation. arXiv 2015.* arXiv preprint arXiv:1505.04597, 2019.
