# Supplementary material for: Live-dead assay on unlabeled cells using phase imaging with computational specificity
Source: Nat Commun. 2022 Feb 7;13:713. doi: 10.1038/s41467-022-28214-x (PMC8821584; doi:10.1038/s41467-022-28214-x)
Supplement: Supplementary file 2 — Reporting Summary [file 41467_2022_28214_MOESM2_ESM.pdf]

nature research

Corresponding author(s): Gabriel Popescu

Last updated by author(s): Dec 19, 2021

Reporting Summary

Nature Research wishes to improve the reproducibility of the work that we publish. This form provides structure for consistency and transparency in reporting. For further information on Nature Research policies, see our Editorial Policies and the Editorial Policy Checklist.

Statistics

For all statistical analyses, confirm that the following items are present in the figure legend, table legend, main text, or Methods section.

n/a	Confirmed	
☐	☒	The exact sample size (*n*) for each experimental group/condition, given as a discrete number and unit of measurement
☐	☒	A statement on whether measurements were taken from distinct samples or whether the same sample was measured repeatedly
☐	☒	The statistical test(s) used AND whether they are one- or two-sided<br>*Only common tests should be described solely by name; describe more complex techniques in the Methods section.*
☒	☐	A description of all covariates tested
☒	☐	A description of any assumptions or corrections, such as tests of normality and adjustment for multiple comparisons
☐	☒	A full description of the statistical parameters including central tendency (e.g. means) or other basic estimates (e.g. regression coefficient) AND variation (e.g. standard deviation) or associated estimates of uncertainty (e.g. confidence intervals)
☐	☒	For null hypothesis testing, the test statistic (e.g. F, t, r) with confidence intervals, effect sizes, degrees of freedom and P value noted<br>*Give P values as exact values whenever suitable.*
☒	☐	For Bayesian analysis, information on the choice of priors and Markov chain Monte Carlo settings
☒	☐	For hierarchical and complex designs, identification of the appropriate level for tests and full reporting of outcomes
☒	☐	Estimates of effect sizes (e.g. Cohen's d, Pearson's r), indicating how they were calculated

Our web collection on statistics for biologists contains articles on many of the points above.

Software and code

Policy information about availability of computer code

Data collection	Data Collection is made via CellVista SLIM Pro, a product by Phi Optics.
Data analysis	The MATLAB script and representative images for semantic segmentation maps are provided as Supplemental Material. The trained E-U-Net models along with sample testing images are available for download at https://github.com/shenghh2020/label-free-viability-assay.

For manuscripts utilizing custom algorithms or software that are central to the research but not yet described in published literature, software must be made available to editors and reviewers. We strongly encourage code deposition in a community repository (e.g. GitHub). See the Nature Research guidelines for submitting code & software for further information.

Data

Policy information about availability of data

All manuscripts must include a data availability statement. This statement should provide the following information, where applicable:

- Accession codes, unique identifiers, or web links for publicly available datasets
- A list of figures that have associated raw data
- A description of any restrictions on data availability

An example set of the SLIM images and corresponding viability maps used in this study is deposited in the GitHub repository “shenghh2015/label-free-viability-assay” [https://github.com/shenghh2015/label-free-viability-assay]. The complete dataset is not deposited due to its massive size, but available from the corresponding author upon request.

Field-specific reporting

Please select the one below that is the best fit for your research. If you are not sure, read the appropriate sections before making your selection.

☒ Life sciences ☐ Behavioural & social sciences ☐ Ecological, evolutionary & environmental sciences

For a reference copy of the document with all sections, see nature.com/documents/nr-reporting-summary-flat.pdf

Life sciences study design

All studies must disclose on these points even when the disclosure is negative.

Sample size	Images of adherent cell were collected and applied in training a deep neural network (DNN). To obtain reliable trained network, the dataset needs a sufficient instances (> 1000). In this study, a DNN were training on two dataset, and the sample size for each dataset is 1298 and 1824. Sample size was determined by the total number of images collected in the imaging experiments.
Data exclusions	In the step of network training, images of empty backgrounds were excluded and not used in training.
Replication	We repeated the imaging experiment 4 times to capture the variability among different batches. All collected image sequences were combined to form a dataset for training and testing
Randomization	During time-lapse acquisition, the cellular region to be measured were randomly selected. Before training DNN, we randomly assign the tiles to form training, validation, and testing dataset. Each tile is a time-lapse sequence of measurement.
Blinding	The researchers were blinded to the splitting of the DNN training and validation dataset. For the statistics analysis shown in Fig. 5 e-f. all recorded data were used to produce the results.

Reporting for specific materials, systems and methods

We require information from authors about some types of materials, experimental systems and methods used in many studies. Here, indicate whether each material, system or method listed is relevant to your study. If you are not sure if a list item applies to your research, read the appropriate section before selecting a response.

Materials & experimental systems

n/a	Involved in the study
☐	☒ Antibodies
☐	☒ Eukaryotic cell lines
☒	☐ Palaeontology and archaeology
☒	☐ Animals and other organisms
☒	☐ Human research participants
☒	☐ Clinical data
☒	☐ Dual use research of concern

Methods

n/a	Involved in the study
☒	☐ ChIP-seq
☒	☐ Flow cytometry
☒	☐ MRI-based neuroimaging

Antibodies

Antibodies used	ReadyProbes Cell Viability Reagents manufactured by ThermoFisher were purchased and used in this study. This product has two fluorescent labeled antibody stains that combine with nucleus of live and dead cells.
Validation	*Describe the validation of each primary antibody for the species and application, noting any validation statements on the manufacturer's website, relevant citations, antibody profiles in online databases, or data provided in the manuscript.*

Eukaryotic cell lines

Policy information about cell lines

Cell line source(s)	In this study, HeLa cervical cancer cells (ATCC CCL-2TM) and Chinese hamster ovary (CHO-K1 ATCC CCL-61 TM) cells were purchased from ATCC and kept frozen in liquid nitrogen.
Authentication	Cell lines used were not authenticated.
Mycoplasma contamination	The cell lines were not tested for mycoplasma contamination.
Commonly misidentified lines (See ICLAC register)	No commonly misidentified cell lines were used in the study.
