# Supplementary material for: Live-dead assay on unlabeled cells using phase imaging with computational specificity
Source: Nat Commun. 2022 Feb 7;13:713. doi: 10.1038/s41467-022-28214-x (PMC8821584; doi:10.1038/s41467-022-28214-x)
Supplement: Supplementary file 4 — Description of Additional Supplementary Files [file 41467_2022_28214_MOESM4_ESM.pdf]

Title: Supplementary Movie 1:
Description: Animation of HeLa cells with viability reagents measured by SLIM (left), corresponding PICS prediction (middle), and standard viability assay (right)

Title: Supplementary Movie 2:
Description: Animation of HeLa cells without staining reagents measured by SLIM (left), and corresponding PICS prediction (right)

Title: Supplementary Movie 3:
Description: Realtime SLIM measurement and PICS viability prediction

Title: Supplementary Software
Description: The Supplementary Software contains the MATLAB script used in this study to generate semantic segmentation maps, along with a few input images for testing. The instructions and computer requirements for running the program is documented in Readme.txt included in the file.
