# Supplementary figures and images for: Live-dead assay on unlabeled cells using phase imaging with computational specificity
Source: Nat Commun. 2022 Feb 7;13:713. doi: 10.1038/s41467-022-28214-x (PMC8821584; doi:10.1038/s41467-022-28214-x)

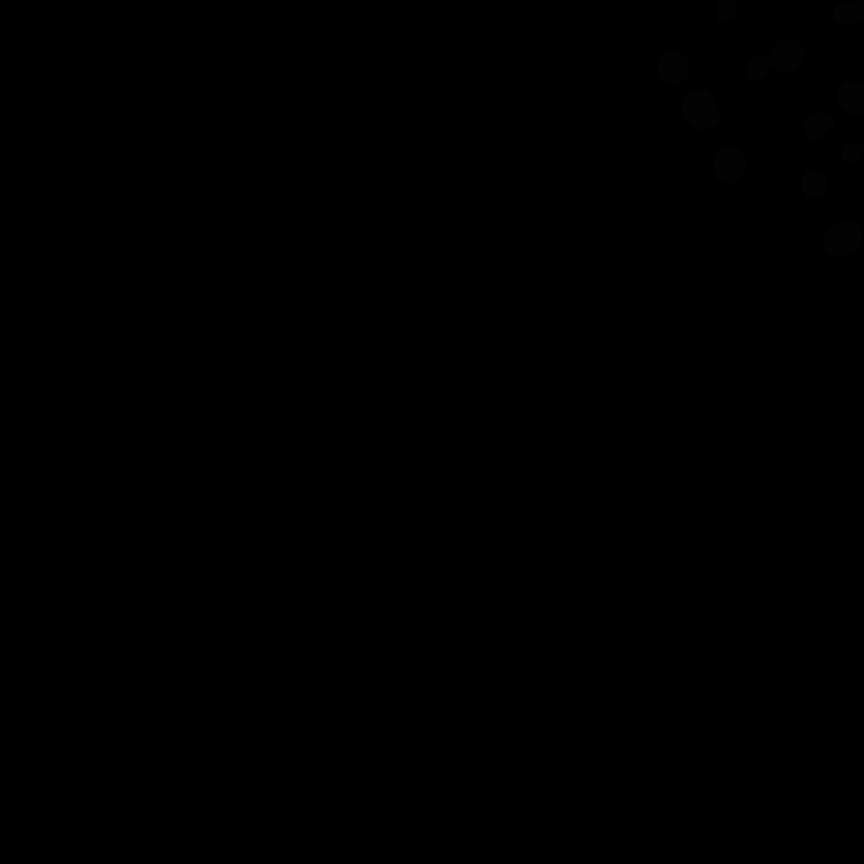

Supplement: Supplementary file 8 — Supplementary Software [file 41467_2022_28214_MOESM8_ESM.zip › data/Ground Truth/f0_t4_i0_ch0_c2_r2_z0_mSeg.tif]

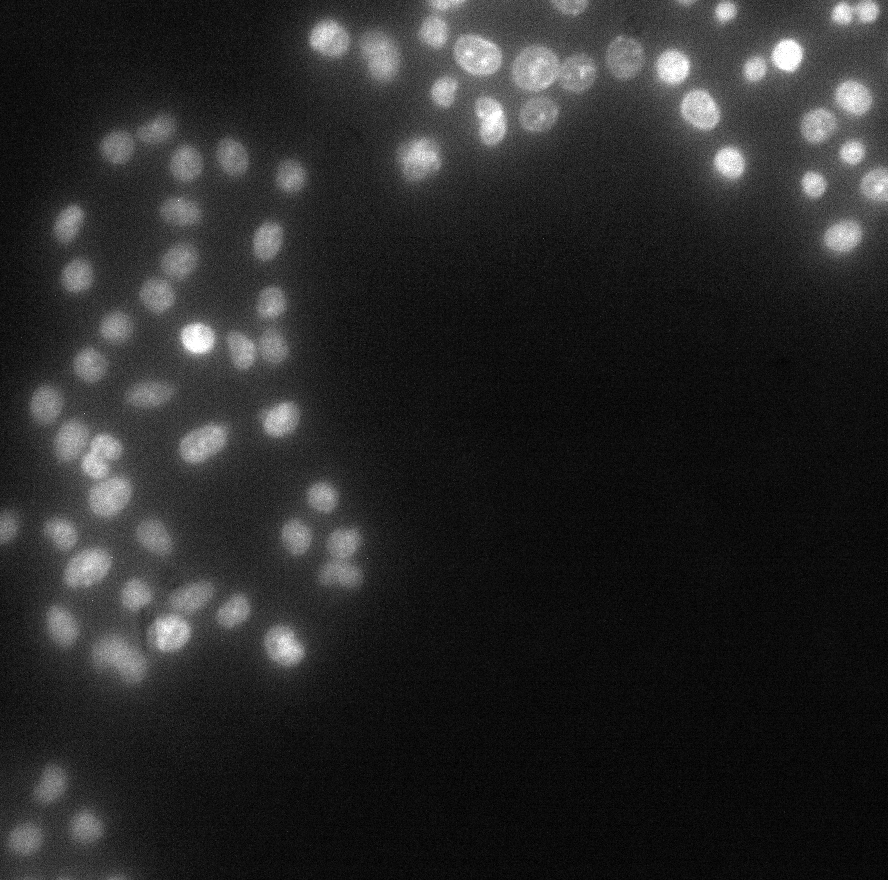

Supplement: Supplementary file 8 — Supplementary Software [file 41467_2022_28214_MOESM8_ESM.zip › data/NucBlue/f0_t4_i0_ch1_c2_r2_z0_mDAPI0.tif]

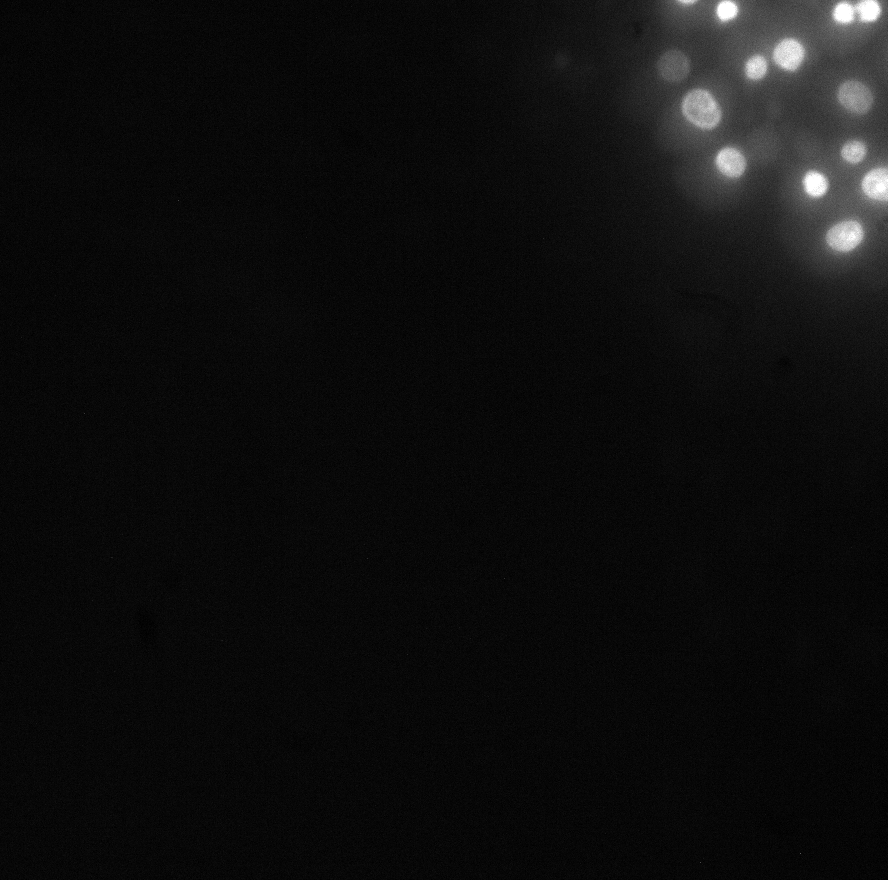

Supplement: Supplementary file 8 — Supplementary Software [file 41467_2022_28214_MOESM8_ESM.zip › data/NucGreen/f0_t4_i0_ch2_c2_r2_z0_mFITC0.tif]

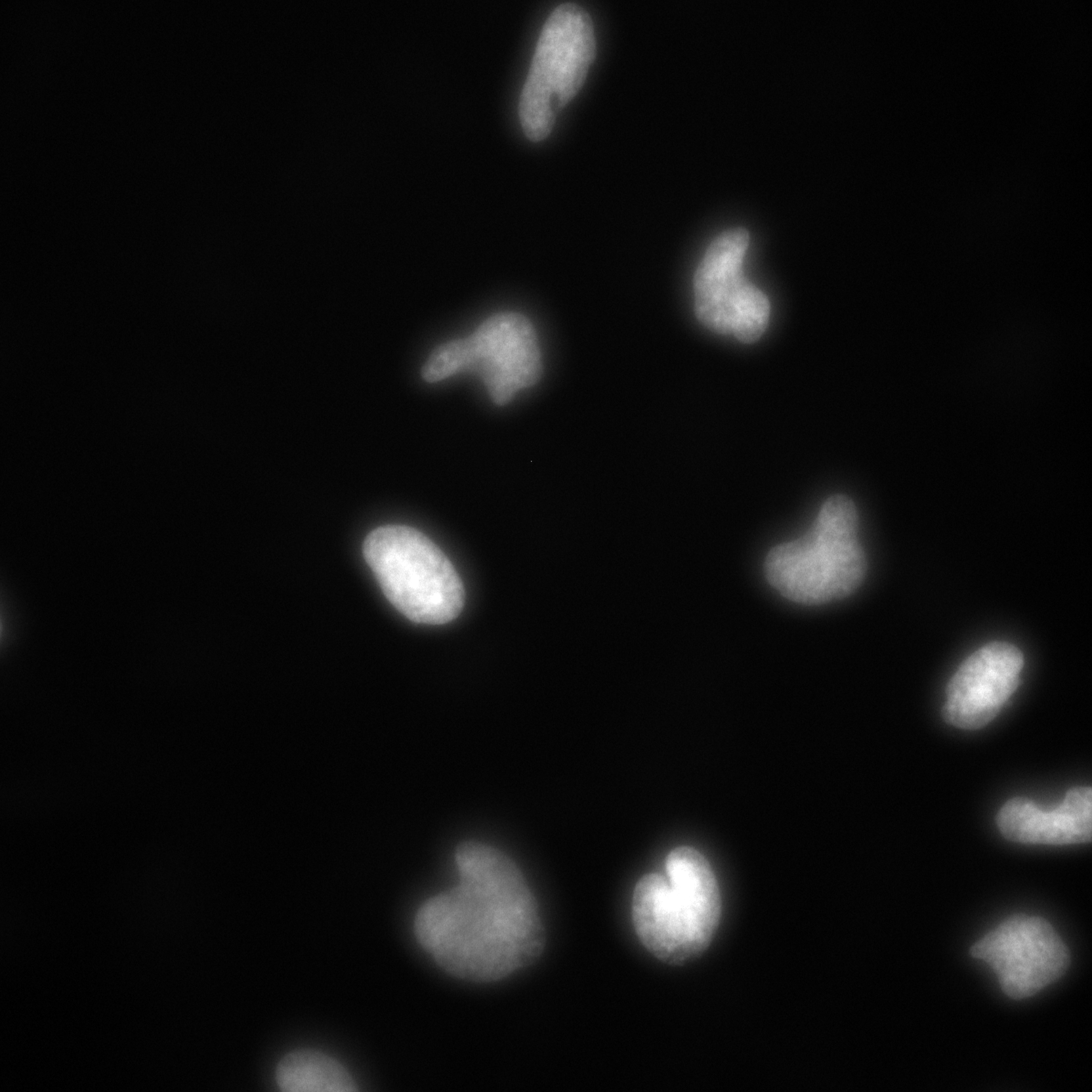

Supplement: Supplementary file 9 — Source Data Files [file 41467_2022_28214_MOESM9_ESM.zip › Source Data Files/Fig1b_1.tif]

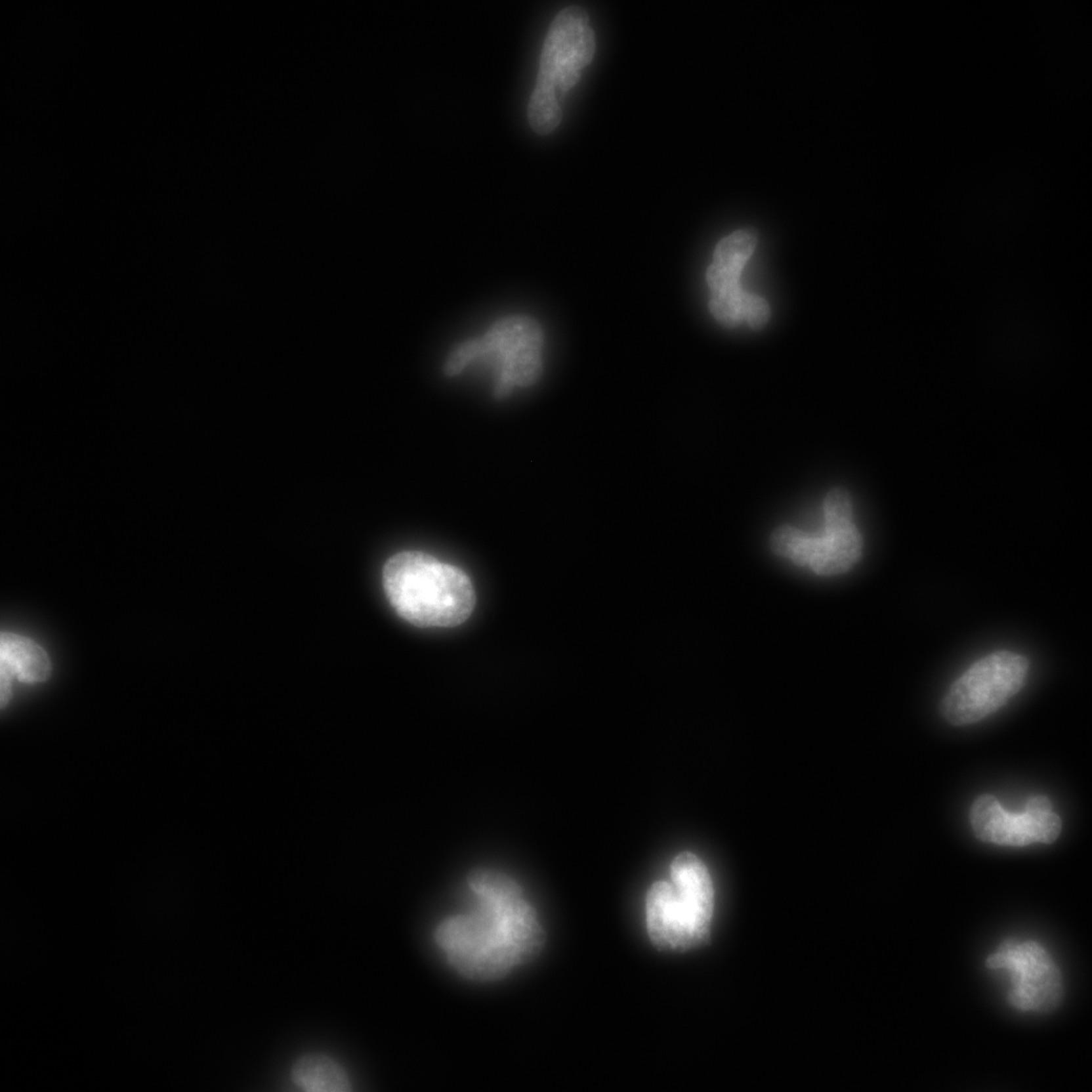

Supplement: Supplementary file 9 — Source Data Files [file 41467_2022_28214_MOESM9_ESM.zip › Source Data Files/Fig1b_2.tif]

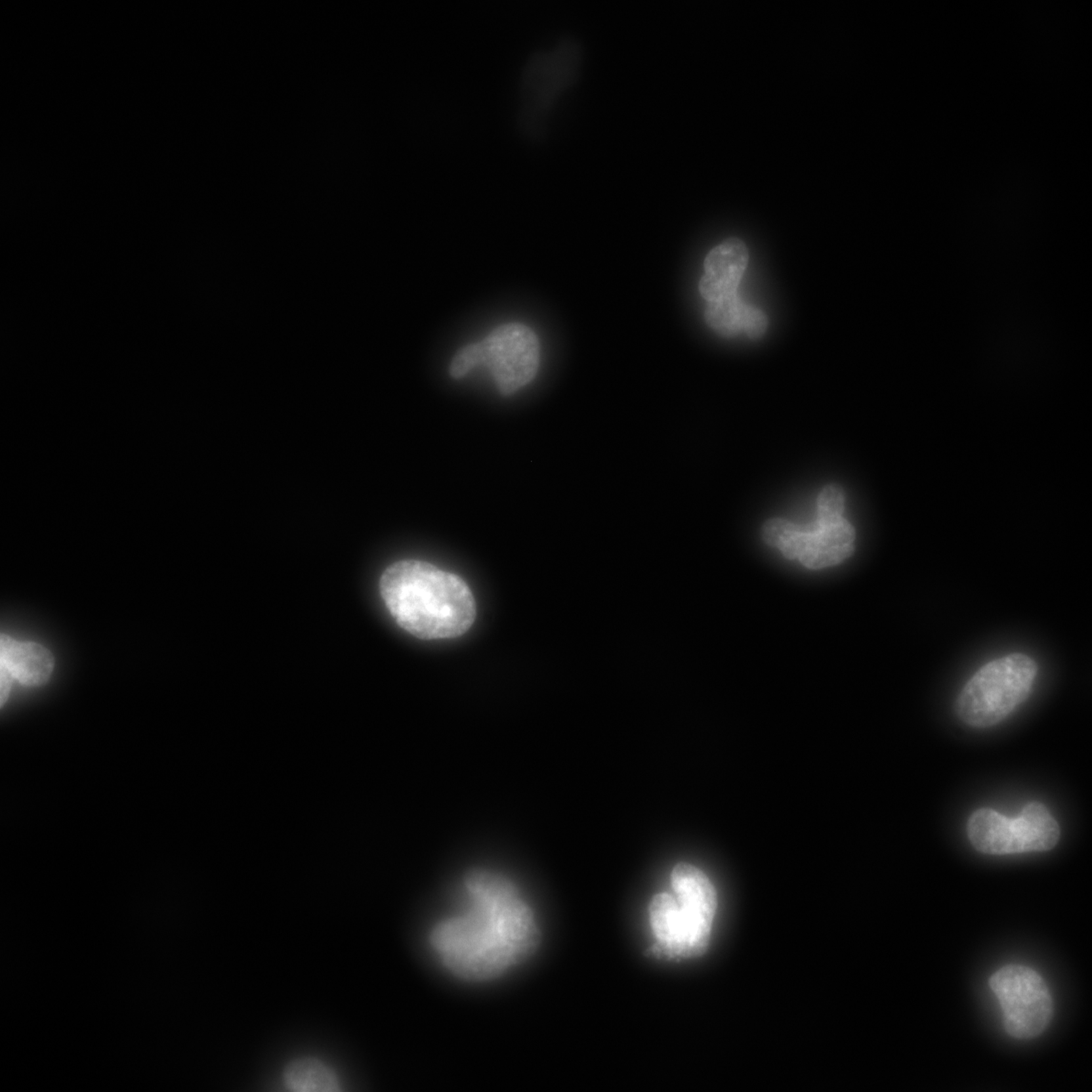

Supplement: Supplementary file 9 — Source Data Files [file 41467_2022_28214_MOESM9_ESM.zip › Source Data Files/Fig1b_3.tif]

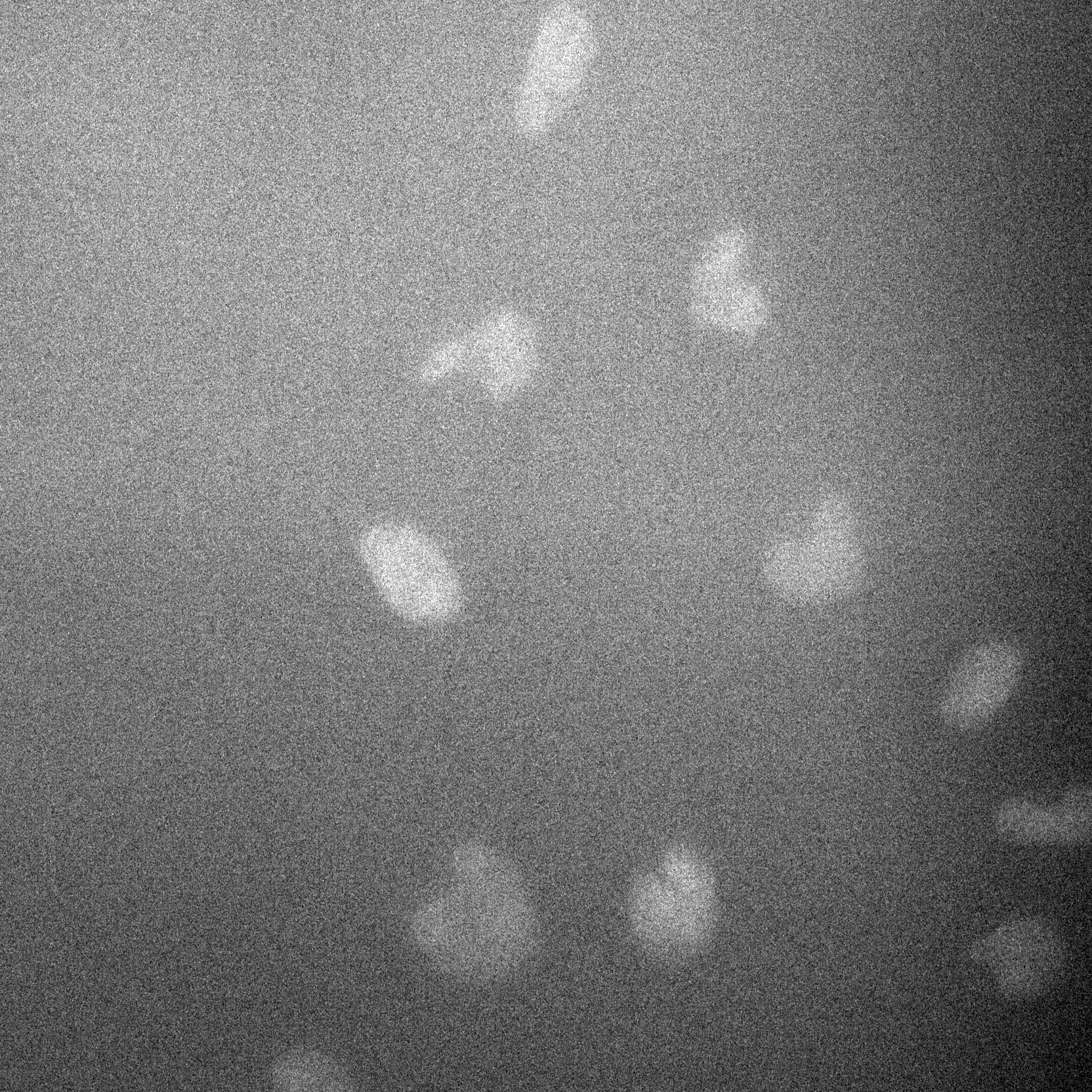

Supplement: Supplementary file 9 — Source Data Files [file 41467_2022_28214_MOESM9_ESM.zip › Source Data Files/Fig1c_1.tif]

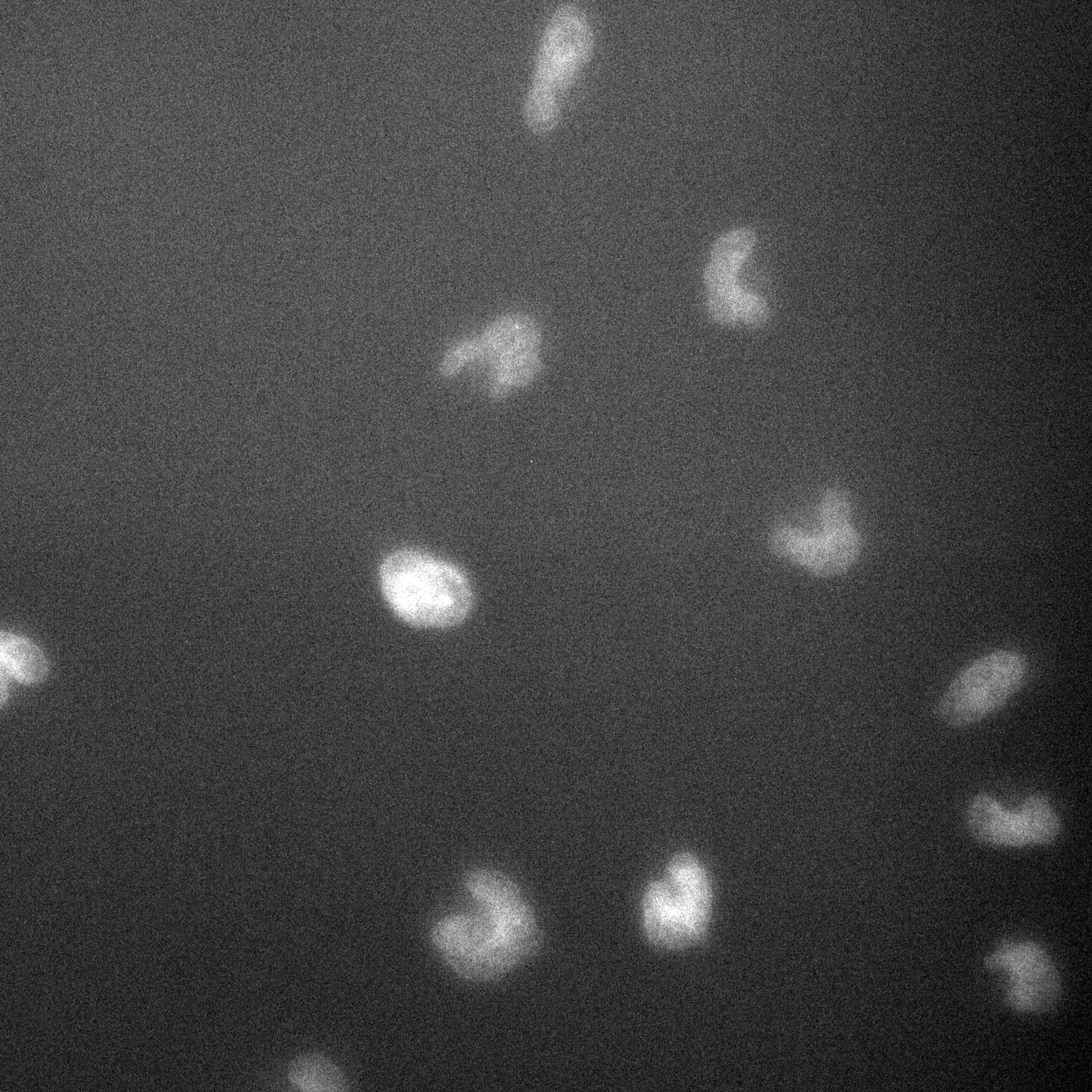

Supplement: Supplementary file 9 — Source Data Files [file 41467_2022_28214_MOESM9_ESM.zip › Source Data Files/Fig1c_2.tif]

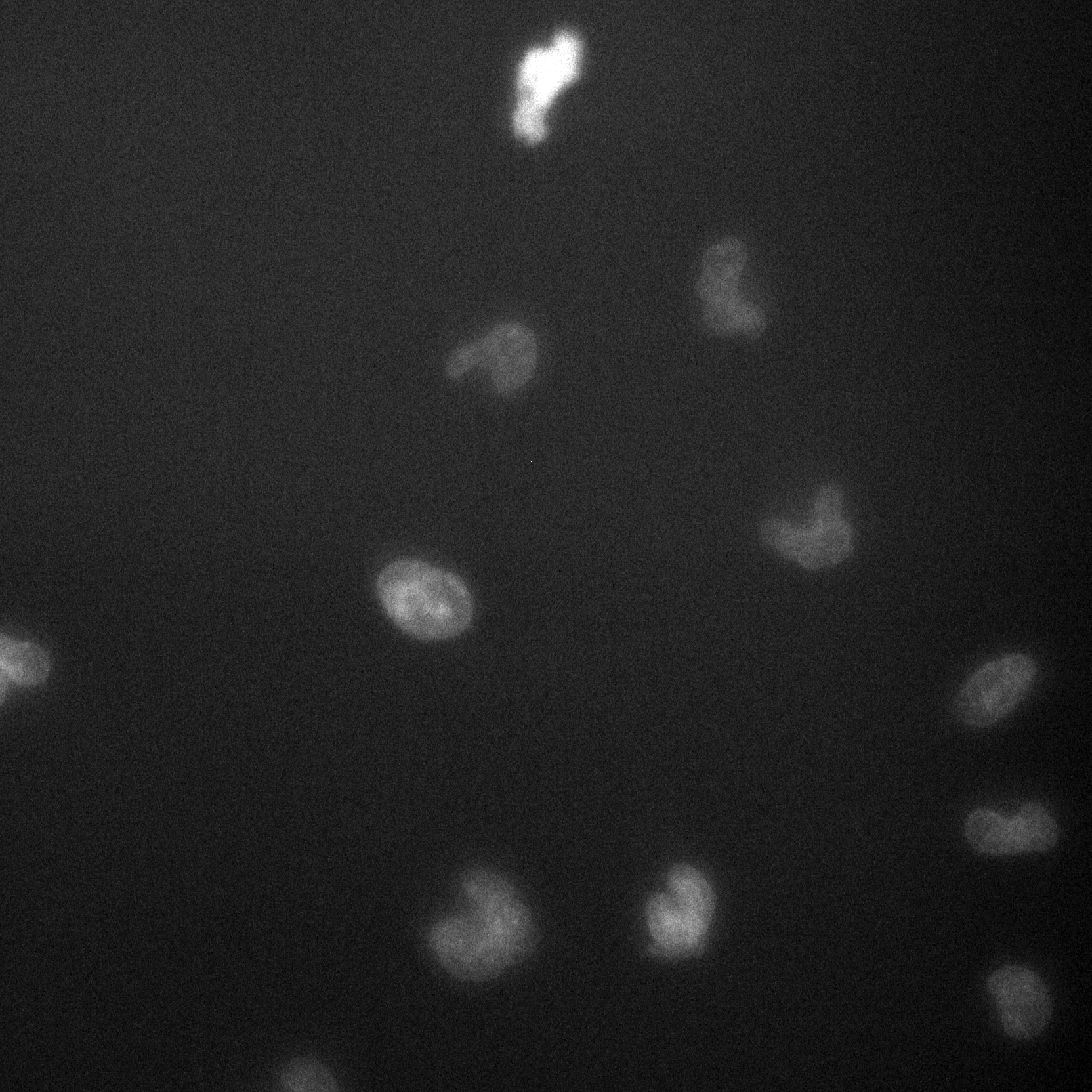

Supplement: Supplementary file 9 — Source Data Files [file 41467_2022_28214_MOESM9_ESM.zip › Source Data Files/Fig1c_3.tif]

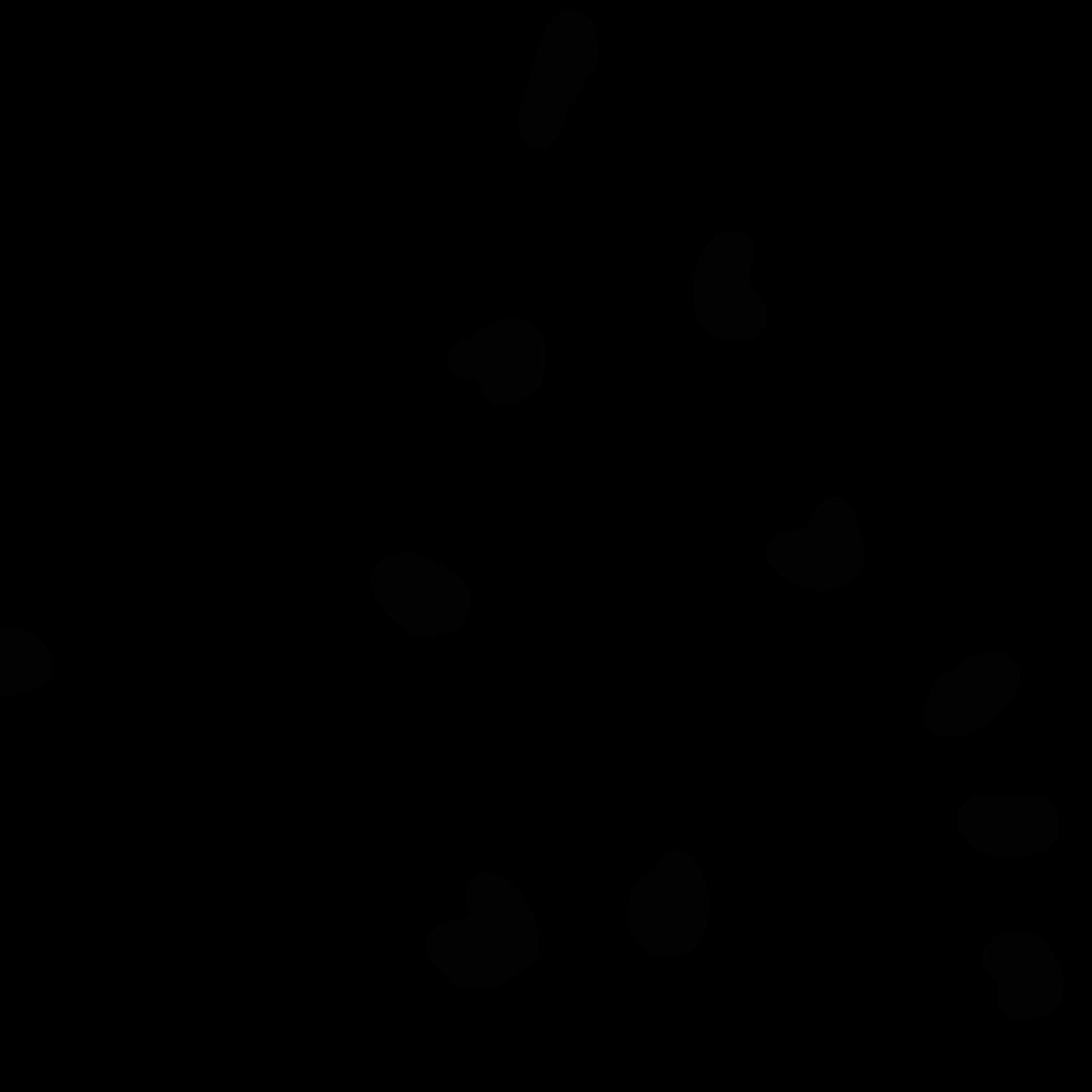

Supplement: Supplementary file 9 — Source Data Files [file 41467_2022_28214_MOESM9_ESM.zip › Source Data Files/Fig1d_1.tif]

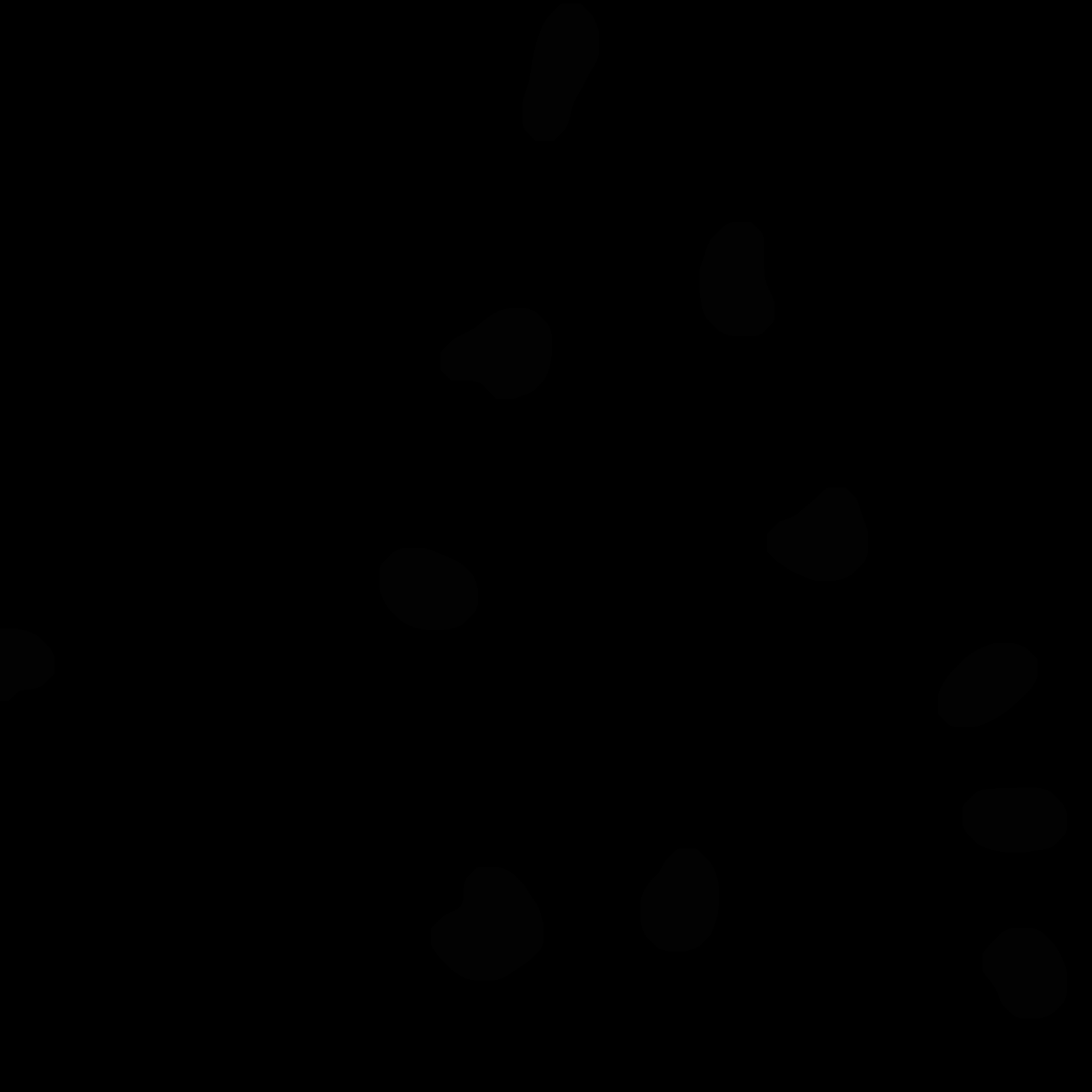

Supplement: Supplementary file 9 — Source Data Files [file 41467_2022_28214_MOESM9_ESM.zip › Source Data Files/Fig1d_2.tif]

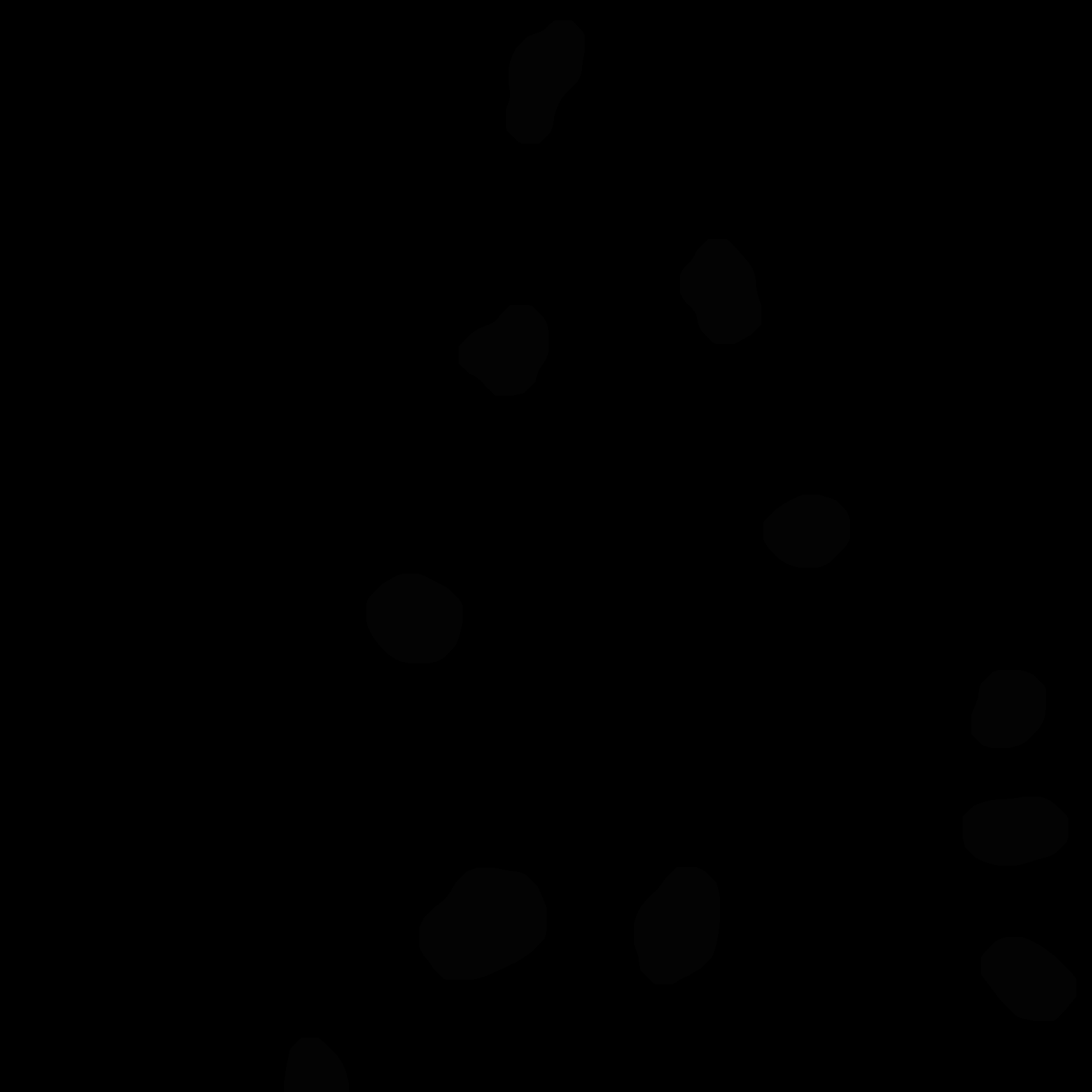

Supplement: Supplementary file 9 — Source Data Files [file 41467_2022_28214_MOESM9_ESM.zip › Source Data Files/Fig1d_3.tif]

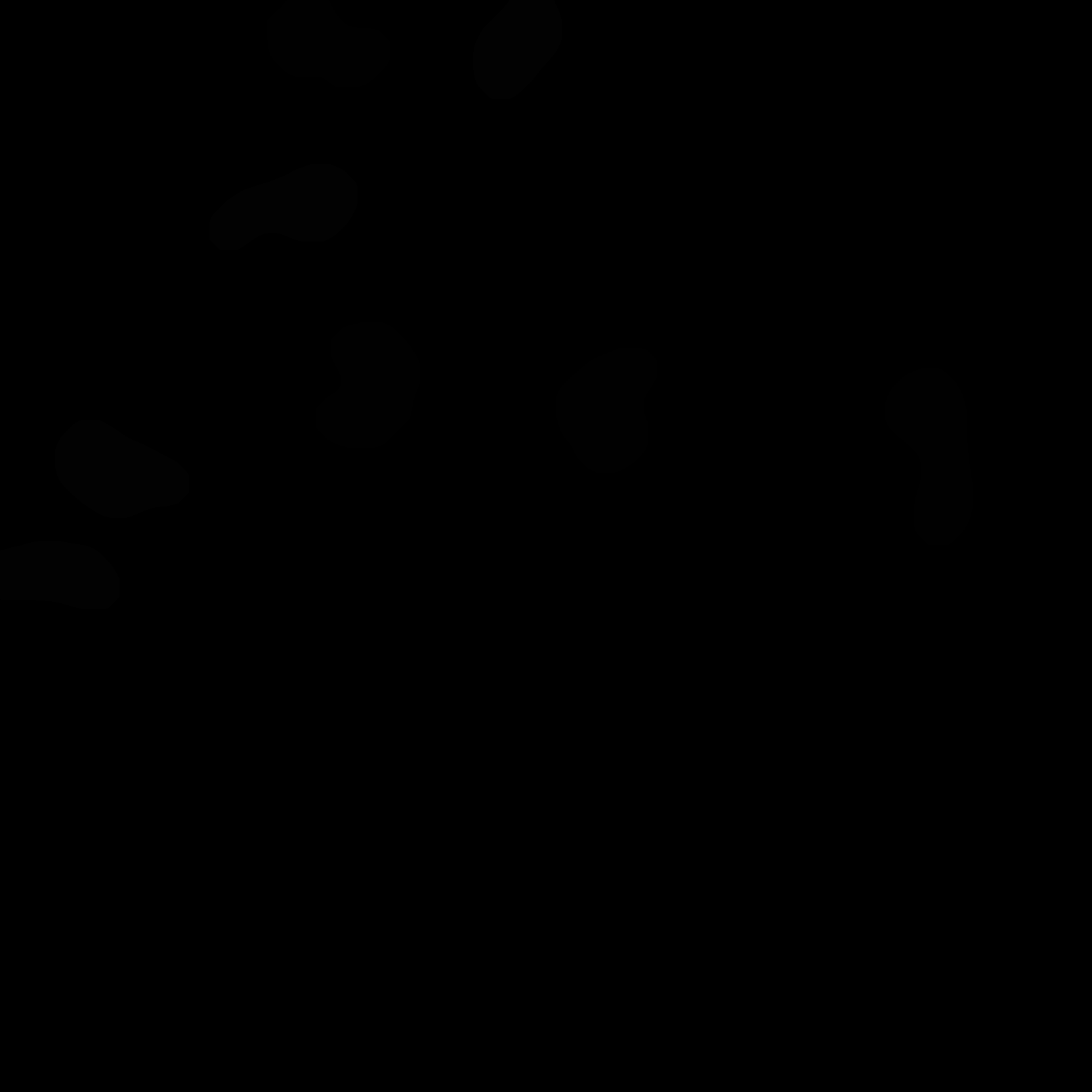

Supplement: Supplementary file 9 — Source Data Files [file 41467_2022_28214_MOESM9_ESM.zip › Source Data Files/Fig3b_1.tif]

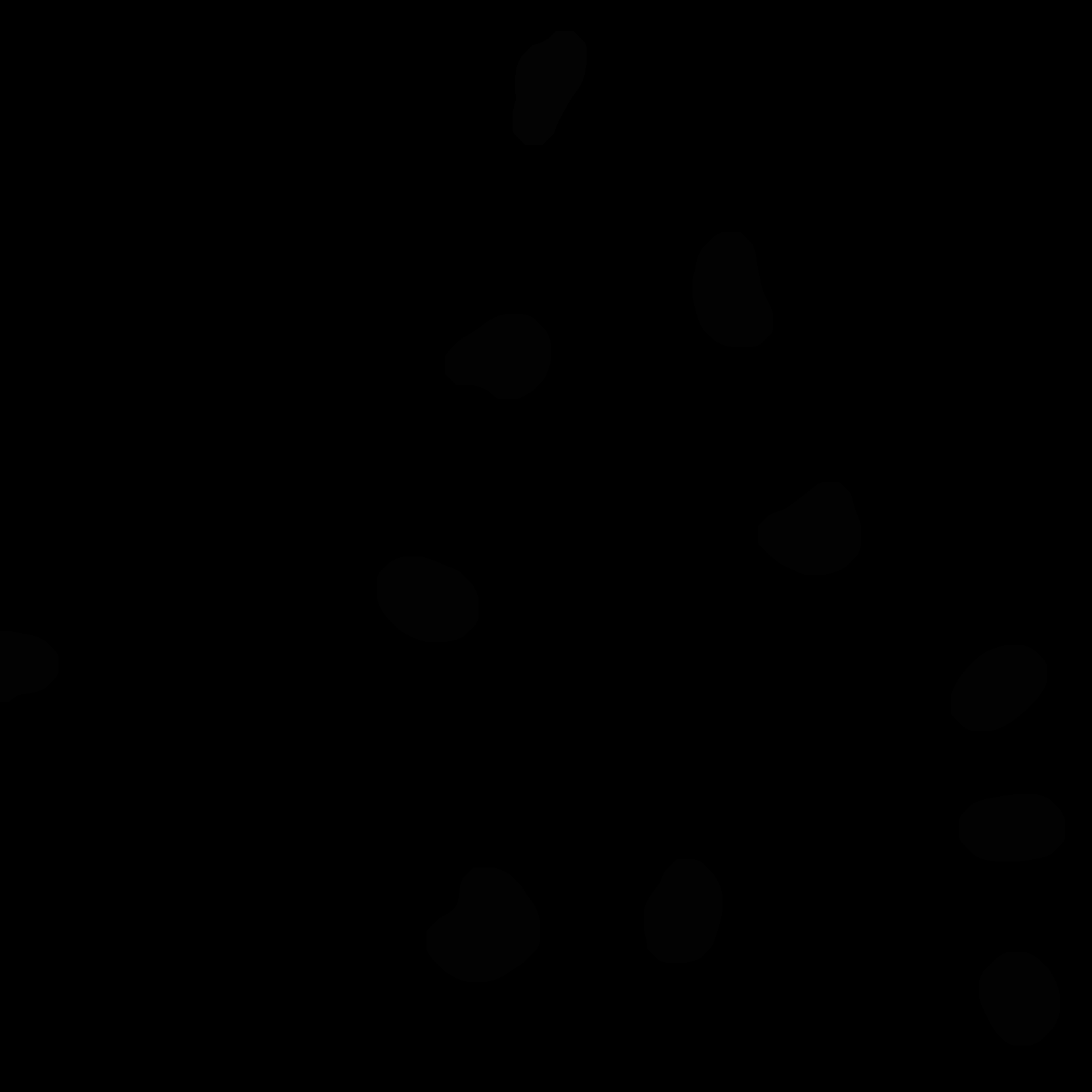

Supplement: Supplementary file 9 — Source Data Files [file 41467_2022_28214_MOESM9_ESM.zip › Source Data Files/Fig3b_2.tif]

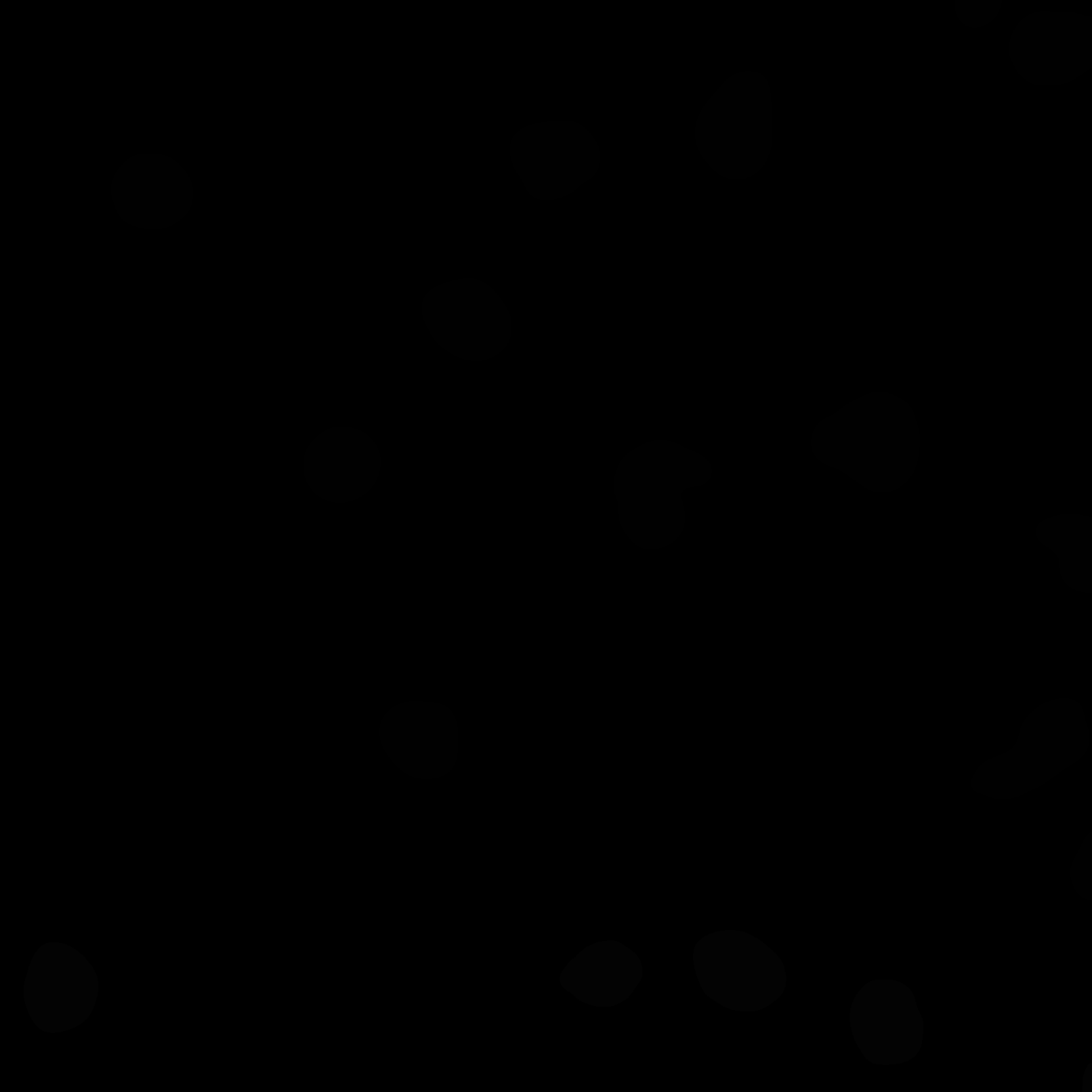

Supplement: Supplementary file 9 — Source Data Files [file 41467_2022_28214_MOESM9_ESM.zip › Source Data Files/Fig3b_3.tif]

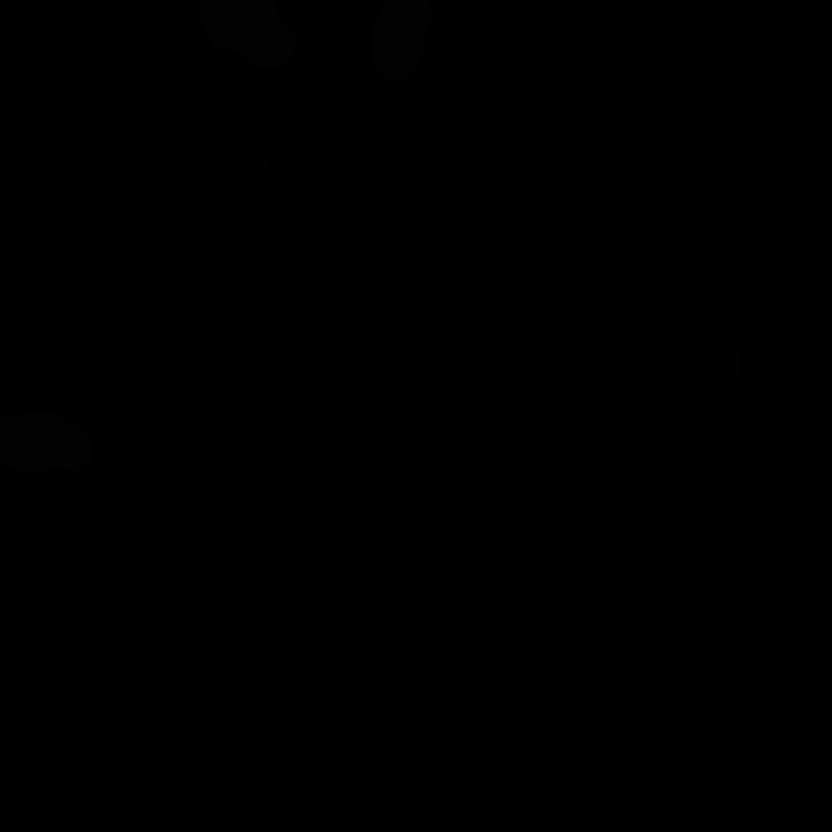

Supplement: Supplementary file 9 — Source Data Files [file 41467_2022_28214_MOESM9_ESM.zip › Source Data Files/Fig3c_1.png]

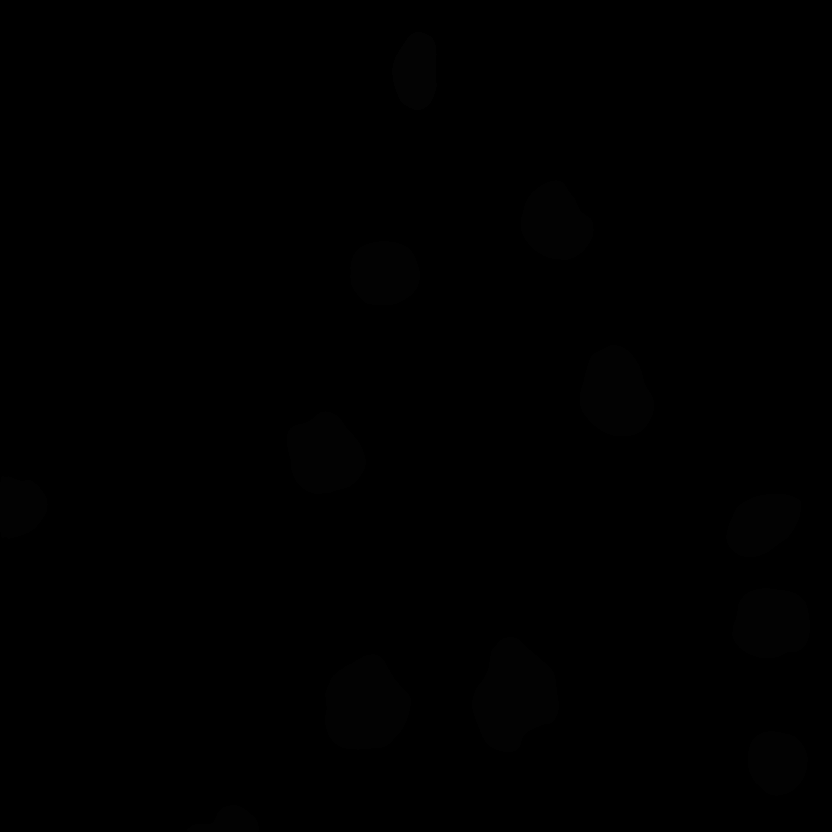

Supplement: Supplementary file 9 — Source Data Files [file 41467_2022_28214_MOESM9_ESM.zip › Source Data Files/Fig3c_2.png]

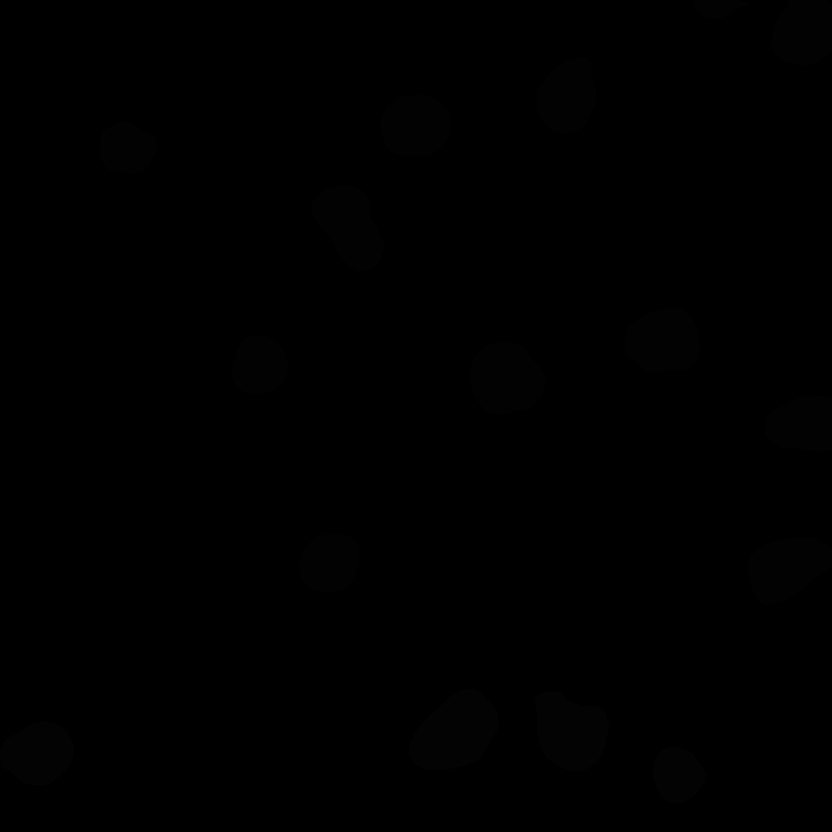

Supplement: Supplementary file 9 — Source Data Files [file 41467_2022_28214_MOESM9_ESM.zip › Source Data Files/Fig3c_3.png]

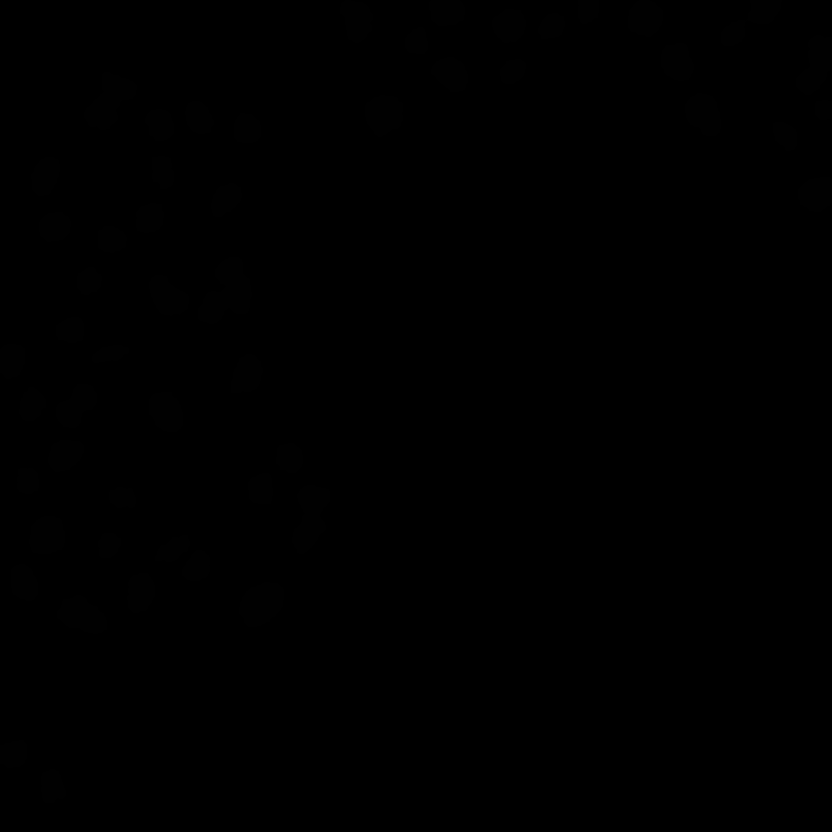

Supplement: Supplementary file 9 — Source Data Files [file 41467_2022_28214_MOESM9_ESM.zip › Source Data Files/Fig4b_1.tif]

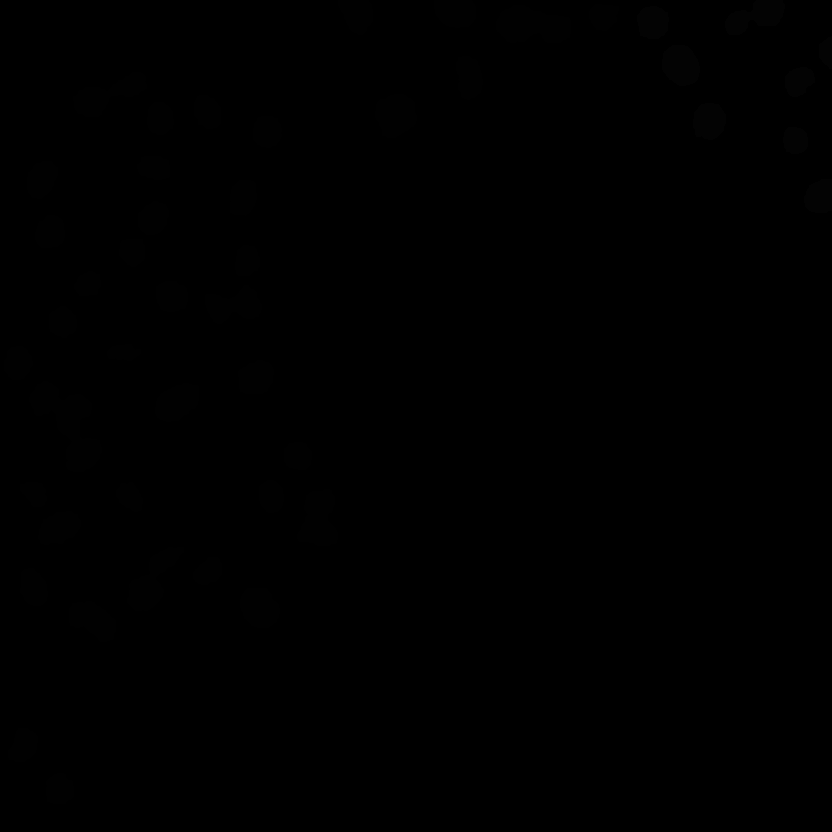

Supplement: Supplementary file 9 — Source Data Files [file 41467_2022_28214_MOESM9_ESM.zip › Source Data Files/Fig4b_2.tif]

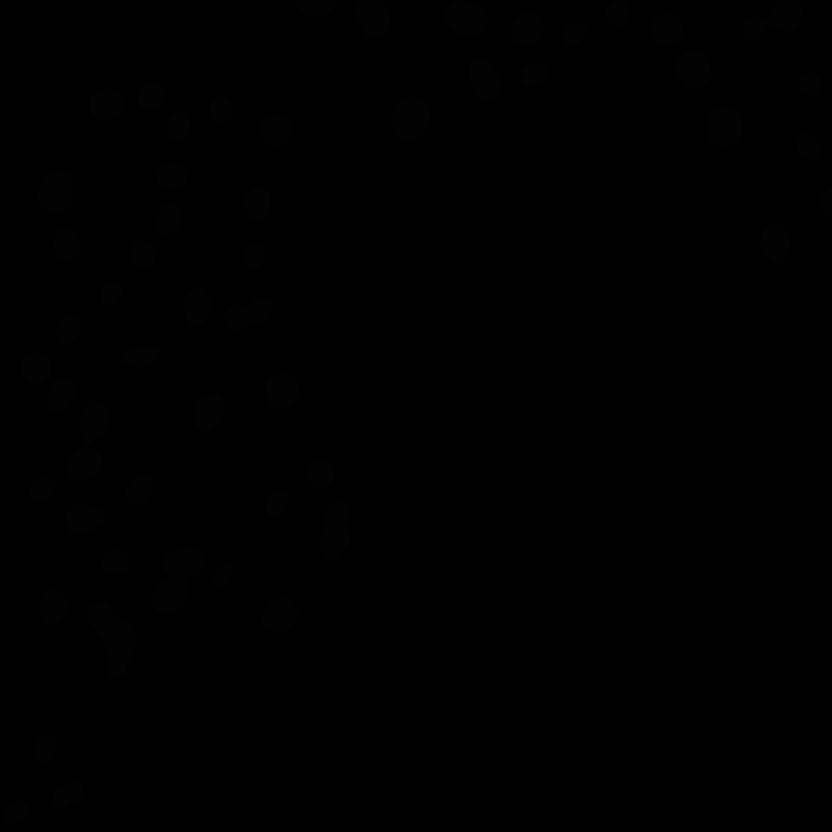

Supplement: Supplementary file 9 — Source Data Files [file 41467_2022_28214_MOESM9_ESM.zip › Source Data Files/Fig4b_3.tif]

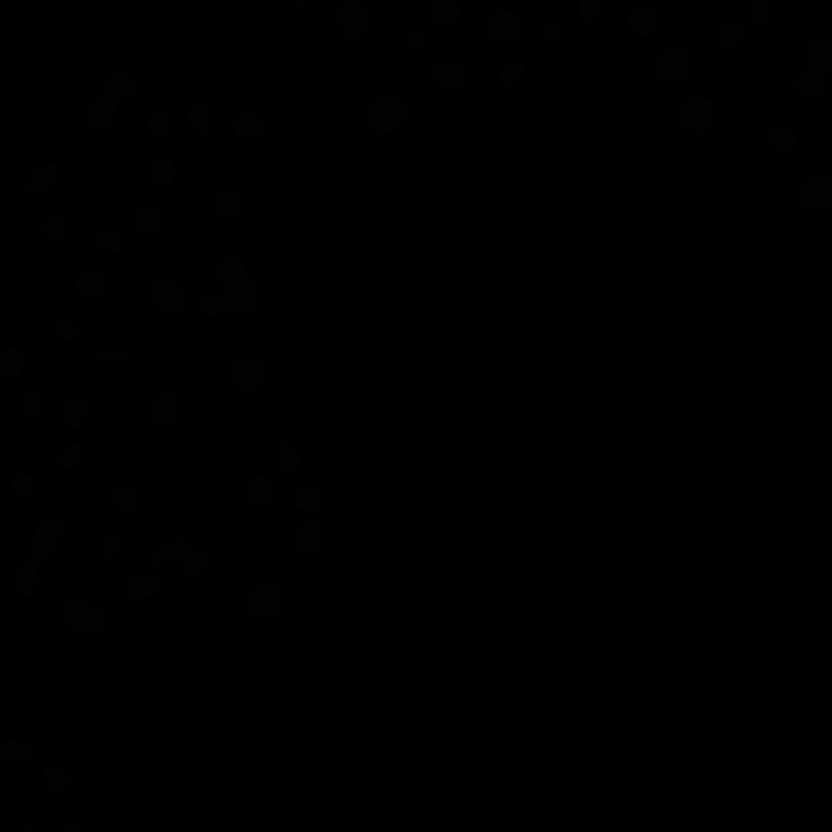

Supplement: Supplementary file 9 — Source Data Files [file 41467_2022_28214_MOESM9_ESM.zip › Source Data Files/Fig4c_1.tif]

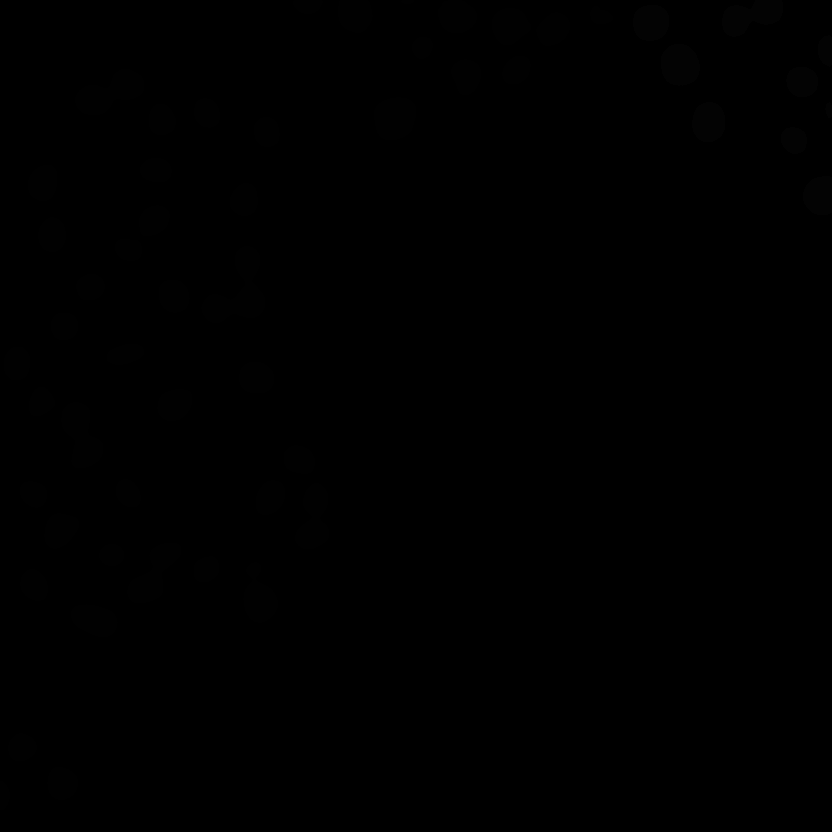

Supplement: Supplementary file 9 — Source Data Files [file 41467_2022_28214_MOESM9_ESM.zip › Source Data Files/Fig4c_2.tif]

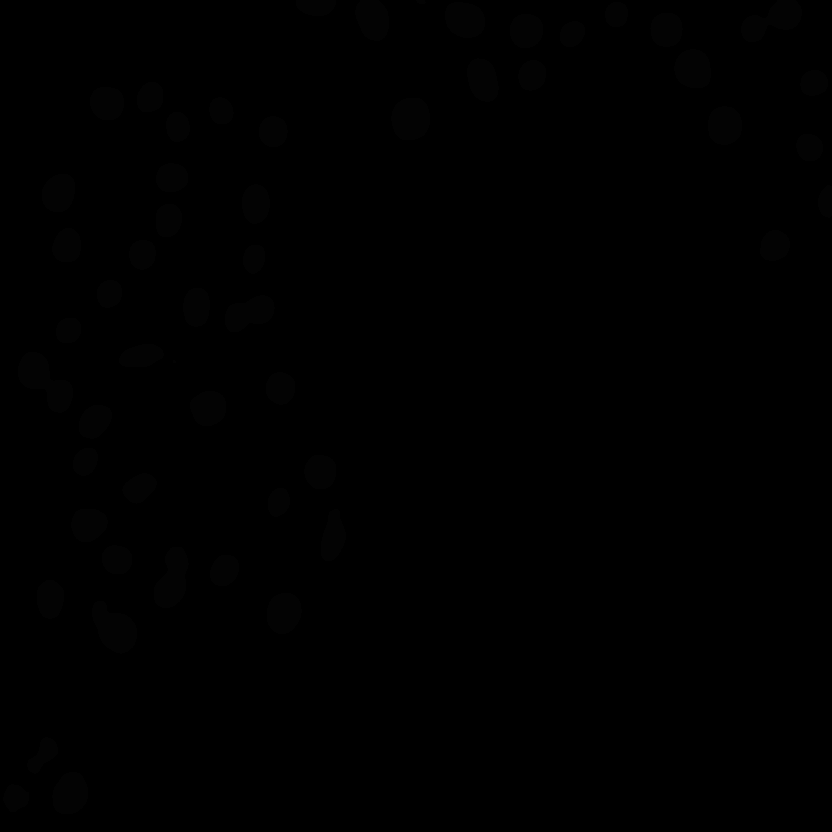

Supplement: Supplementary file 9 — Source Data Files [file 41467_2022_28214_MOESM9_ESM.zip › Source Data Files/Fig4c_3.tif]

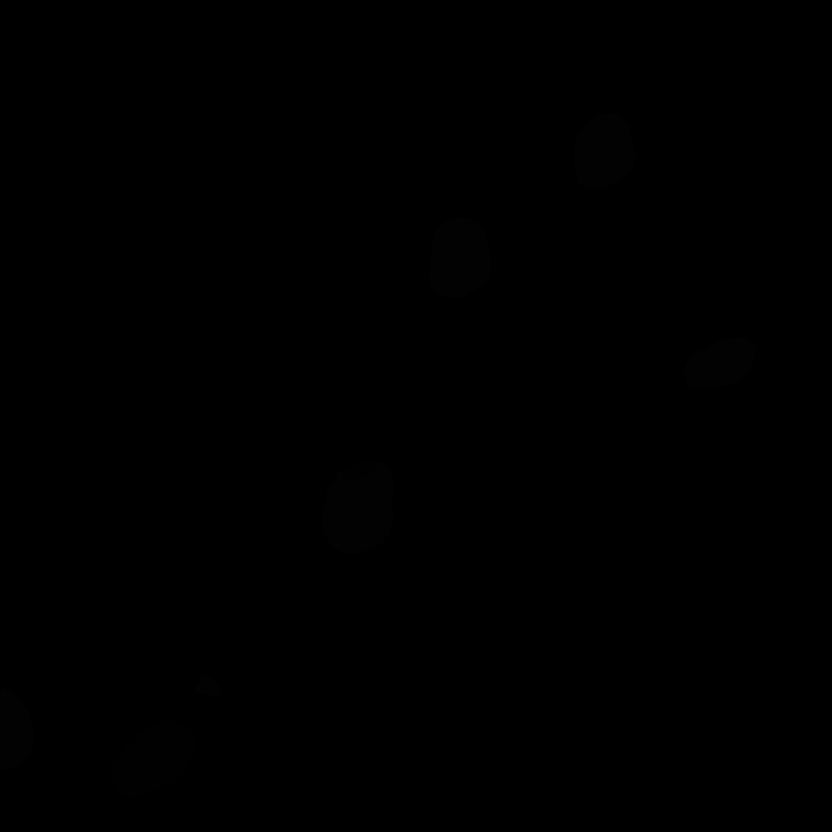

Supplement: Supplementary file 9 — Source Data Files [file 41467_2022_28214_MOESM9_ESM.zip › Source Data Files/Fig5b_1.png]

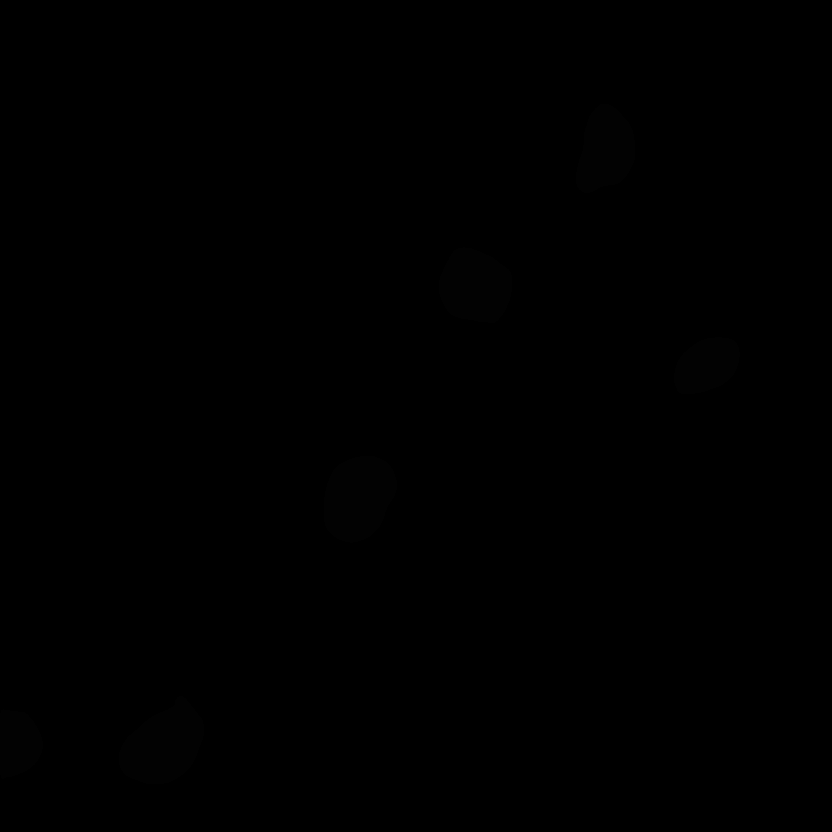

Supplement: Supplementary file 9 — Source Data Files [file 41467_2022_28214_MOESM9_ESM.zip › Source Data Files/Fig5b_2.png]

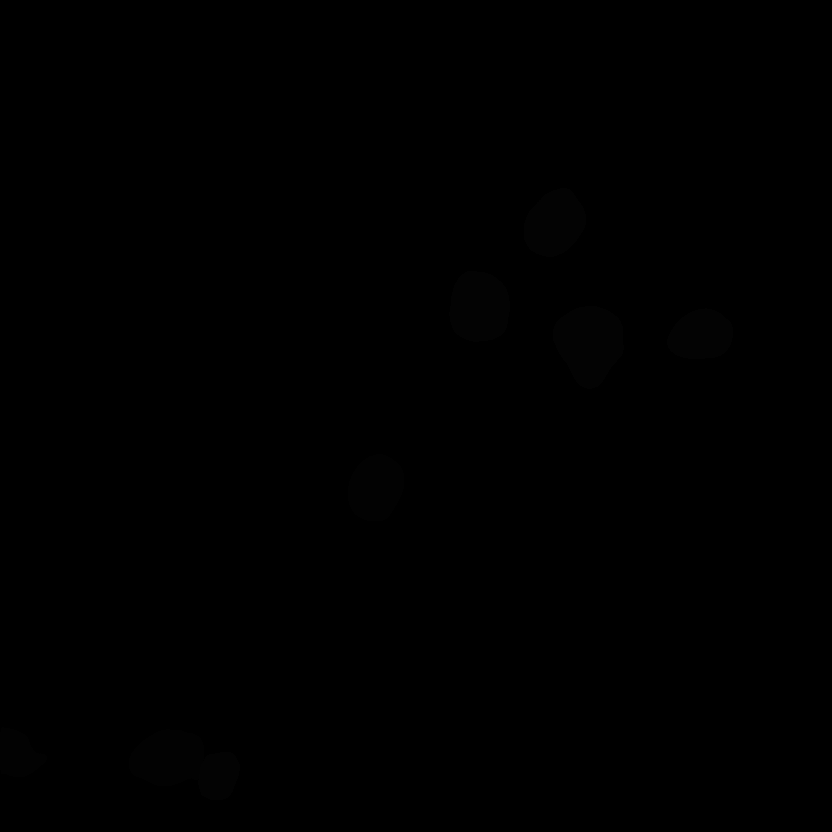

Supplement: Supplementary file 9 — Source Data Files [file 41467_2022_28214_MOESM9_ESM.zip › Source Data Files/Fig5b_3.png]

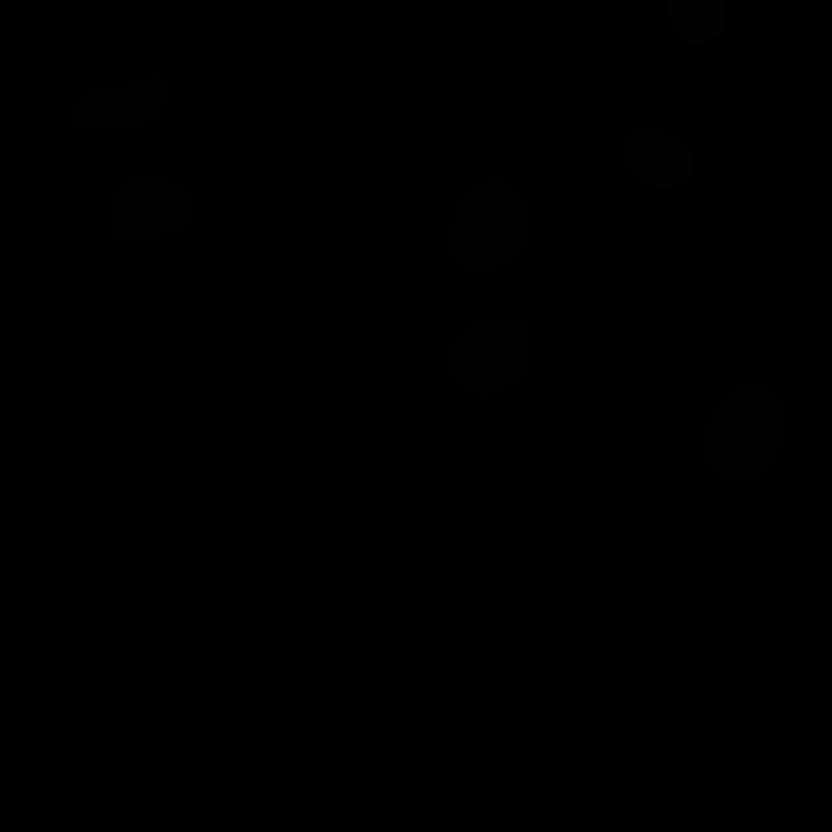

Supplement: Supplementary file 9 — Source Data Files [file 41467_2022_28214_MOESM9_ESM.zip › Source Data Files/Fig5d_1.png]

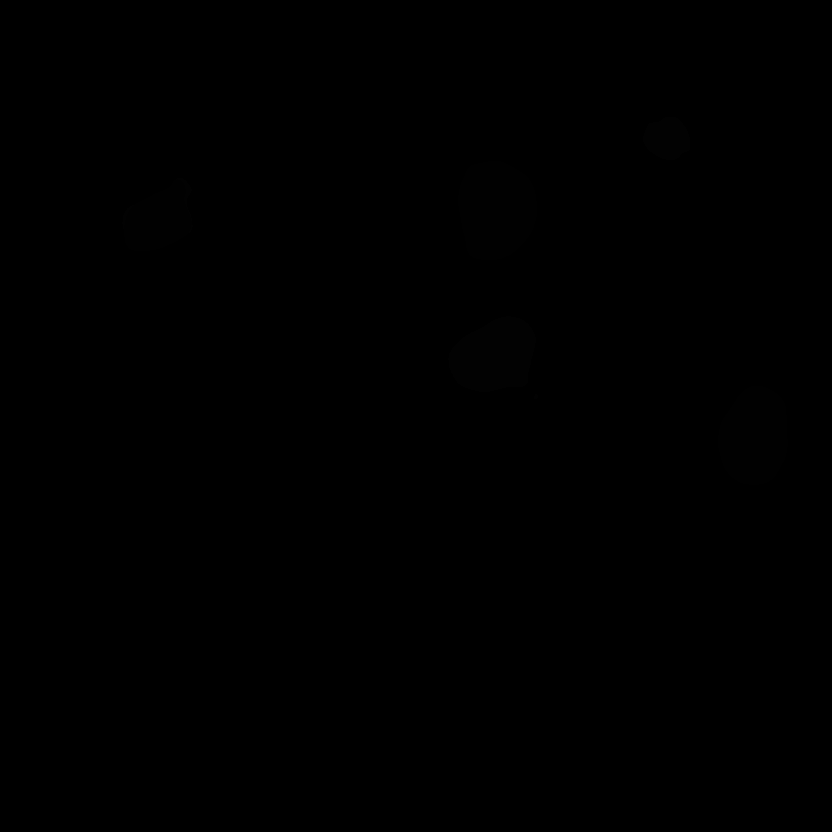

Supplement: Supplementary file 9 — Source Data Files [file 41467_2022_28214_MOESM9_ESM.zip › Source Data Files/Fig5d_2.png]

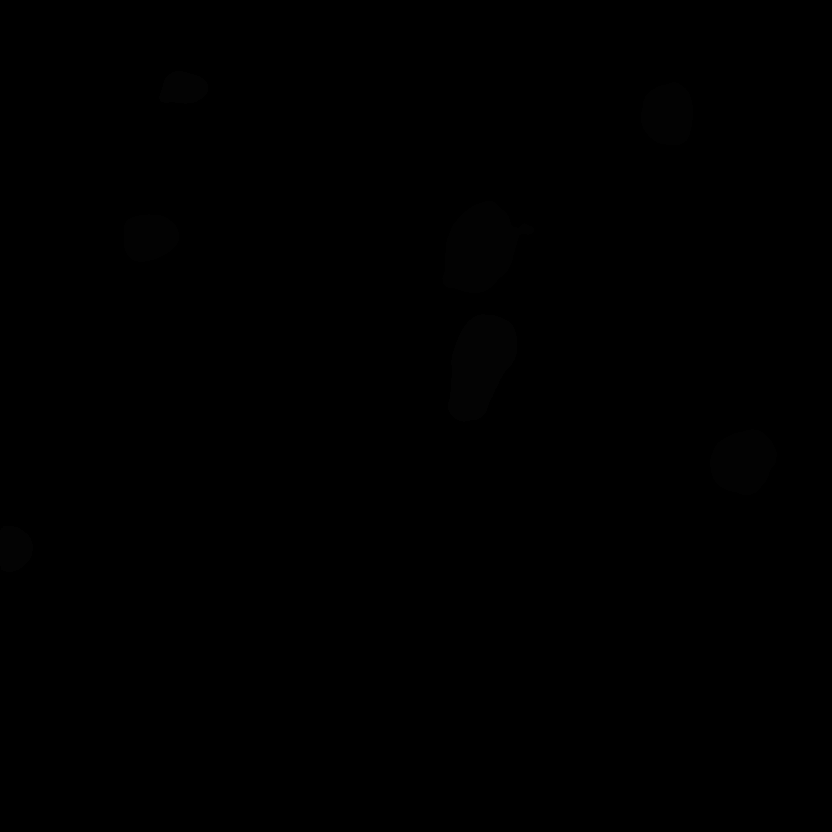

Supplement: Supplementary file 9 — Source Data Files [file 41467_2022_28214_MOESM9_ESM.zip › Source Data Files/Fig5d_3.png]
